# Risk factors for calcification in chronic pancreatitis: a systematic review and meta-analysis

**DOI:** 10.3389/fmed.2025.1703088

**Published:** 2025-11-19

**Authors:** Orsolya Eperjesi, Jázmin Németh, Anett Rancz, Karen Krisztina Fazekas, Brigitta Teutsch, Renáta Papp, Peter Hegyi, Stefania Bunduc

**Affiliations:** 1 Centre for Translational Medicine, Semmelweis University, Budapest, Hungary; 2 Department of Internal Medicine, Toldy Ferenc Hospital, Cegléd, Hungary; 3 Institute of Pancreatic Diseases, Semmelweis University, Budapest, Hungary; 4 Institute for Translational Medicine, Medical School, University of Pécs, Pécs, Hungary; 5 Department of Radiology, Medical Imaging Centre, Semmelweis University, Budapest, Hungary; 6 Department of Pharmacology and Pharmacotherapy, Semmelweis University, Budapest, Hungary; 7 Center for Pharmacology and Drug Research & Development, Semmelweis University, Budapest, Hungary; 8 Centre of Science and Innovation Vice-rector and Business Development, Semmelweis University, Budapest, Hungary; 9 Translational Pancreatology Research Group, Interdisciplinary Centre of Excellence for Research Development and Innovation, University of Szeged, Szeged, Hungary; 10 Faculty of Medicine, Carol Davila University of Medicine and Pharmacy Bucharest, Bucharest, Romania; 11 Digestive Disease and Liver Transplant Center, Fundeni Clinical Institute, Bucharest, Romania

**Keywords:** alcohol drinking, pancreatic calculi, smoking, tobacco use disorder, alcohol-related disorders

## Abstract

**Background/objectives:**

Pancreatic calcifications are the most common structural change in chronic pancreatitis (CP). They can be associated with increased morbidity and healthcare costs. The factors associated with calcifying CP phenotype are not fully understood. Therefore, we aimed to investigate risk factors for calcification in patients with CP.

**Methods:**

The study protocol was registered in PROSPERO(CRD42024591837). On September 18, 2024, we systematically searched three databases (PubMed, EMBASE, and CENTRAL) for studies reporting factors associated with calcifications in CP. Pooled odds ratios (OR) with 95% confidence intervals (CI) were calculated using a random-effects model.

**Results:**

The systematic search resulted in 10,893 articles, of which 80 eligible studies were identified, covering a total of 31,017 participants. Among risk factors investigated, alcohol consumption (OR = 2.31, CI: 1.80–2.98) and smoking (OR = 2.22, CI: 1.61–3.06) were associated with a twofold increase in odds of calcification when compared to non-smokers and non-drinkers, respectively. The odds of calcification were also 45% higher in alcoholic versus idiopathic CP (OR = 1.45, CI: 1.00–2.11). In contrast, the presence of cystic fibrosis transmembrane conductance regulator (CFTR) mutations did not increase the risk of calcification in CP (OR = 0.43, CI: 0.11–1.66) when compared to CFTR wild-type patients.

**Conclusion:**

Modifiable risk factors, such as alcohol consumption and smoking, were significantly associated with calcifying CP phenotype. It is essential to incorporate smoking cessation and alcohol consumption reduction programs into the standard of care for CP.

## 1 Introduction

Chronic pancreatitis (CP) is a progressive, long-term fibroinflammatory disorder primarily influenced by a complex interplay of environmental and genetic risk factors. Diagnosis relies on the presence of characteristic symptoms and morphological pancreatic alterations, of which calcifications are the most common, affecting approximately 67% of patients. In cases of alcohol-related CP, this rate can be as high as 90% (1, 2). When developed, calcifying CP (CCP) phenotype is often difficult to manage, increasing morbidity and leading to multiple admissions and interventions. The factors associated with increased risk of developing calcification in CP are not fully understood.

Currently, there is no definitive evidence that structural alterations directly precede or are correlated with functional impairment in CP (3). Pancreatic calcifications, particularly those located in the main pancreatic duct, are commonly observed and exacerbate the associated pancreatic exocrine insufficiency (PEI) (4). A significant inverse correlation was observed between the number of parenchymal calcifications and Body Mass Index (BMI) (5).

Although not all patients with pancreatic calcifications require intervention, treatment is indicated in approximately 30–50% of cases, particularly when calcifications lead to main duct obstruction, persistent pain, ductal dilatation, or recurrent pancreatitis (6). Endoscopic approaches, most commonly extracorporeal shock wave lithotripsy (ESWL) followed by stone extraction, are typically the first-line option, especially in patients with a dilated main pancreatic duct and a limited number of stones. Surgical intervention is generally reserved for patients with multiple or large intraductal stones, failure of endoscopic therapy, or associated complications (7).

A recent international consensus guideline has proposed an updated, two-step mechanistic definition of CP that incorporates etiological factors to allow for diagnosis in the fibro-inflammatory stage prior to the appearance of structural changes (8). This approach emphasizes the importance of understanding the relationship between exposure to etiological factors and the pattern of structural changes in CP (8). However, the factors associated with calcification in CP are not well investigated (9). A more detailed understanding of the parameters associated with pancreatic calcification may therefore contribute to understanding the pathophysiology of CP, but also to advancing disease classification (10).

To address this knowledge gap, we conducted a systematic review and meta-analysis to identify and quantify etiological, demographic, and genetic risk factors associated with pancreatic calcification in CP.

## 2 Methods

We present our systematic review and meta-analysis as per the 2020 PRISMA guidelines (Supplementary Table S1) and following the Cochrane Handbook recommendations (11, 12). The study protocol was previously registered in PROSPERO (CRD42024591837) and was strictly followed (13). This study was implemented as part of the Semmelweis University Translational Medicine Systems Education Program (14).

### 2.1 Information sources

Our systematic search was conducted on September 18, 2024. We searched MEDLINE (via PubMed), Embase, and the Cochrane Central Register of Controlled Trials (CENTRAL) databases.

### 2.2 Eligibility criteria

We formulated our clinical questions using the following PFO framework: P—Population (CP, as defined in individual studies), F—Factors (any factors reported in association with calcifications, such as demographics, disease etiology, and mutations), O—Outcome (presence of pancreatic calcification on any imaging). Eligible study designs included randomized controlled trials (RCTs) and observational studies.

### 2.3 Search and selection strategy

Our search key was: Chronic AND pancrea* AND (calcif* OR stone OR lithiasis). Additionally, a backward and forward citation search of eligible articles was performed on November 10, 2024, to identify further potentially relevant publications (15). No filters or other restrictions were applied during the selection process. The retrieved articles were processed using EndNote 20 and the Rayyan web software (16, 17). Following duplicate removal, two independent reviewers(OE, JN) performed the selection based on predefined eligibility criteria, reviewing titles, abstracts, and full texts. Cohen’s kappa coefficient(*κ*) was calculated to assess inter-rater reliability after each selection stage. Disagreements were resolved by a third independent reviewer (AR).

### 2.4 Data collection process

Data were independently collected by two authors(OE, JN), with any disagreements resolved by a third author (AR). A standardized data collection sheet was developed based on input from methodological and clinical experts. We extracted the following information from eligible articles: title, first author, digital object identifier (DOI), year of publication, country of origin, study design, patient demographics, etiology of CP, diagnostic methods for calcification, number of patients with and without calcifying CP phenotype and number of patients experiencing and not experiencing factors reported in association with calcifications. If available, odds ratios (ORs) and corresponding 95% confidence intervals (CIs) for the calcifying CP phenotype in patients experiencing the evaluated risk factors were extracted for inclusion in the pooled analysis.

### 2.5 Synthesis methods

Both qualitative and quantitative data syntheses were performed. A meta-analysis was conducted when at least three studies were available for the outcome of interest. The thresholds for stratification based on alcohol consumption (80 g/day) and cigarette smoking (20 cigarettes/day) were selected as they represented the most frequently reported units across the included studies, allowing for a consistent and comparative analysis. Additionally, they correspond to clinically recognized levels of heavy alcohol use and smoking (i.e., 80 g/day and 20 cigarettes/day, respectively), which are commonly associated with increased pancreatic injury in the literature and thus serve as relevant cut-offs for stratification (18).

The effect size for dichotomous outcomes was measured using the OR. If the OR for the risk factors associated with calcification was not reported in the eligible studies, it was calculated based on the number of patients exposed to the risk factor analyzed and the total number of patients in each group (with and without calcification, respectively). The pooled OR was calculated using the Mantel–Haenszel method when relevant event rates were available, or the inverse variance weighting method when the OR was directly provided in the publications (19). If both unadjusted and adjusted OR (AOR) were included in the same article, we used the unadjusted OR in the analysis and presented the AOR value on the forest plot for visualization. The difference between the means (MD) is used for the effect size measure for continuous outcomes. To calculate the study MDs and pooled MD, we extracted or estimated sample size, mean, and corresponding standard deviation (SD) from each study (in each group separately). Meta-analyses were performed using random-effects models to account for between-study heterogeneity, with heterogeneity quantified using Higgins and Thompson’s *I*^2^ statistic (20). Results were considered statistically significant if the pooled 95% CI did not contain the null value. We summarized the findings of the meta-analysis in forest plots. We reported directly the prediction interval only if the number of studies exceeded seven to allow a meaningful estimation. Small study publication bias was assessed by visual inspection of Funnel-plots. All statistical analyses were calculated with R software (21) using the meta package (22) for basic meta-analysis calculations and plots, and the dmetar package (23) for additional influential analysis calculations and plots.

### 2.6 Risk of bias assessment

The risk of bias was assessed following the recommendations of the Cochrane Collaboration, using the Quality of Prognostic Studies (QUIPS) tool (24). Two independent reviewers(OE, JN) conducted the assessment, and any disagreements were resolved by an independent third reviewer (AR). Publication bias was evaluated through visual inspection of funnel plots, and modified Egger’s test was performed where appropriate.

## 3 Results

### 3.1 Study selection and characteristics

**Figure 1:**
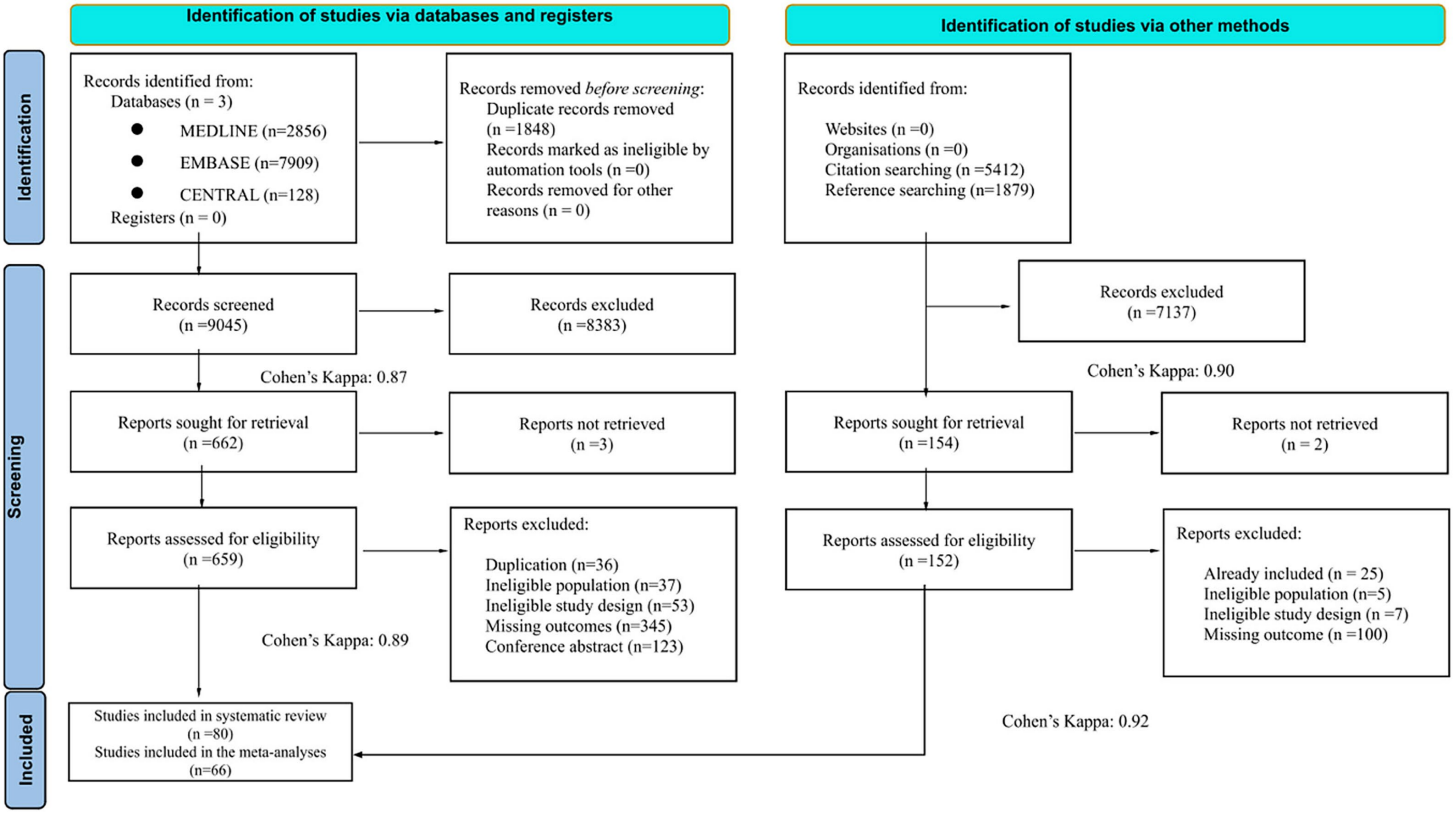
PRISMA flowchart—literature screening process and Cohen’s kappa results.

A total of 10,893 articles were identified by our systematic search. We included 80 studies (31,017 patients) in the systematic review, and 66 studies (27,261 patients) in the pooled analyses. The study selection process is illustrated in the PRISMA flowchart (Figure 1). A summary of baseline characteristics of the studies included is presented in the Supplementary Table S2. The studies included were published between 1979 and 2023, with sample sizes ranging from 19 to 2,153 participants. Of the 80 included studies, study designs comprised 36 prospective, 31 retrospective, and 13 cross-sectional or case–control studies. Studies were conducted across diverse geographic regions, reflecting the heterogeneity of the existing literature.

### 3.2 Demographic and environmental risk factors for the development of calcification in CP

**Figure 2:**
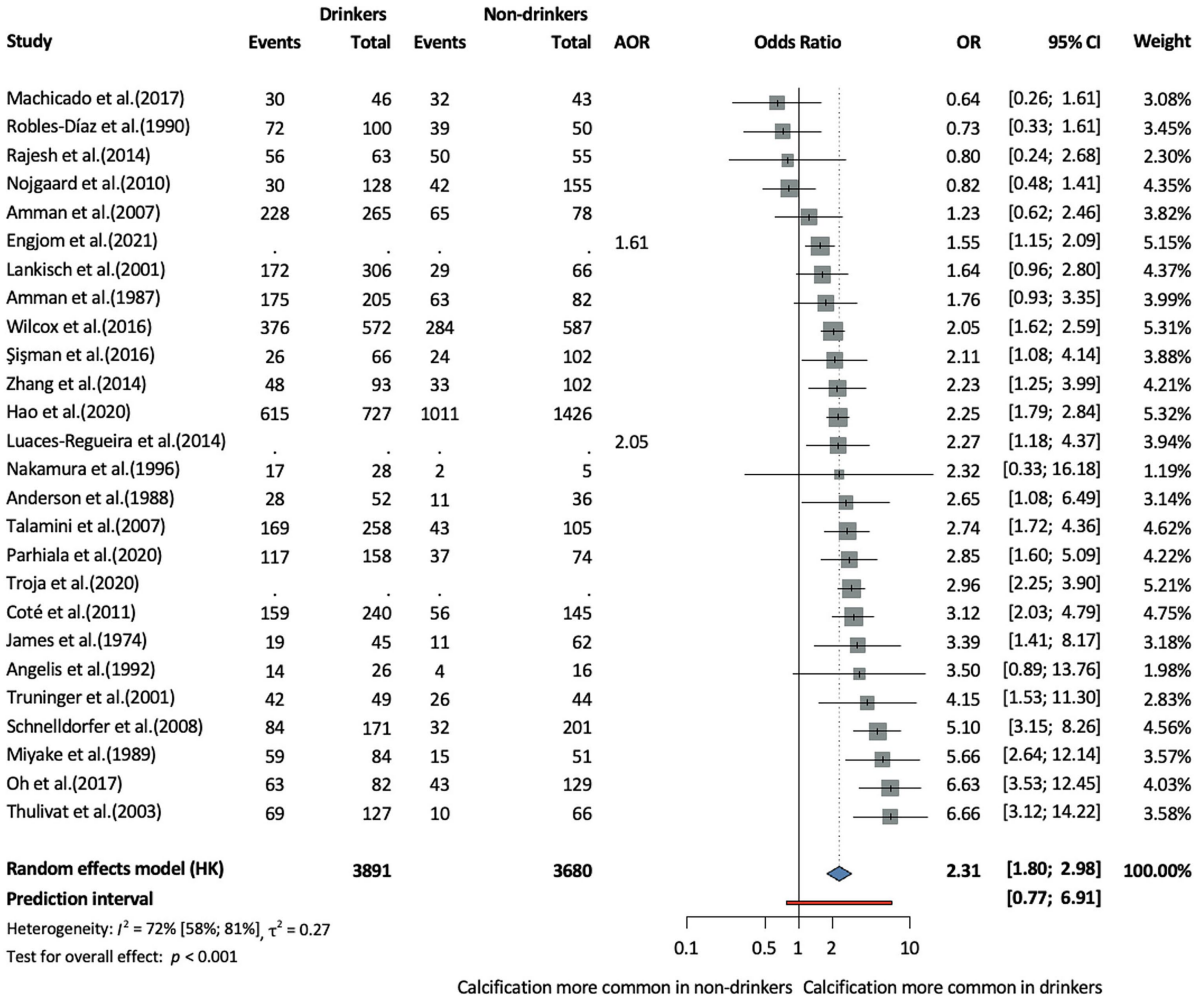
Risk of the calcifying phenotype in chronic pancreatitis in drinkers compared to non-drinkers (AOR, adjusted odds ratio; CI, confidence interval, OR, odds ratio).

**Figure 3:**
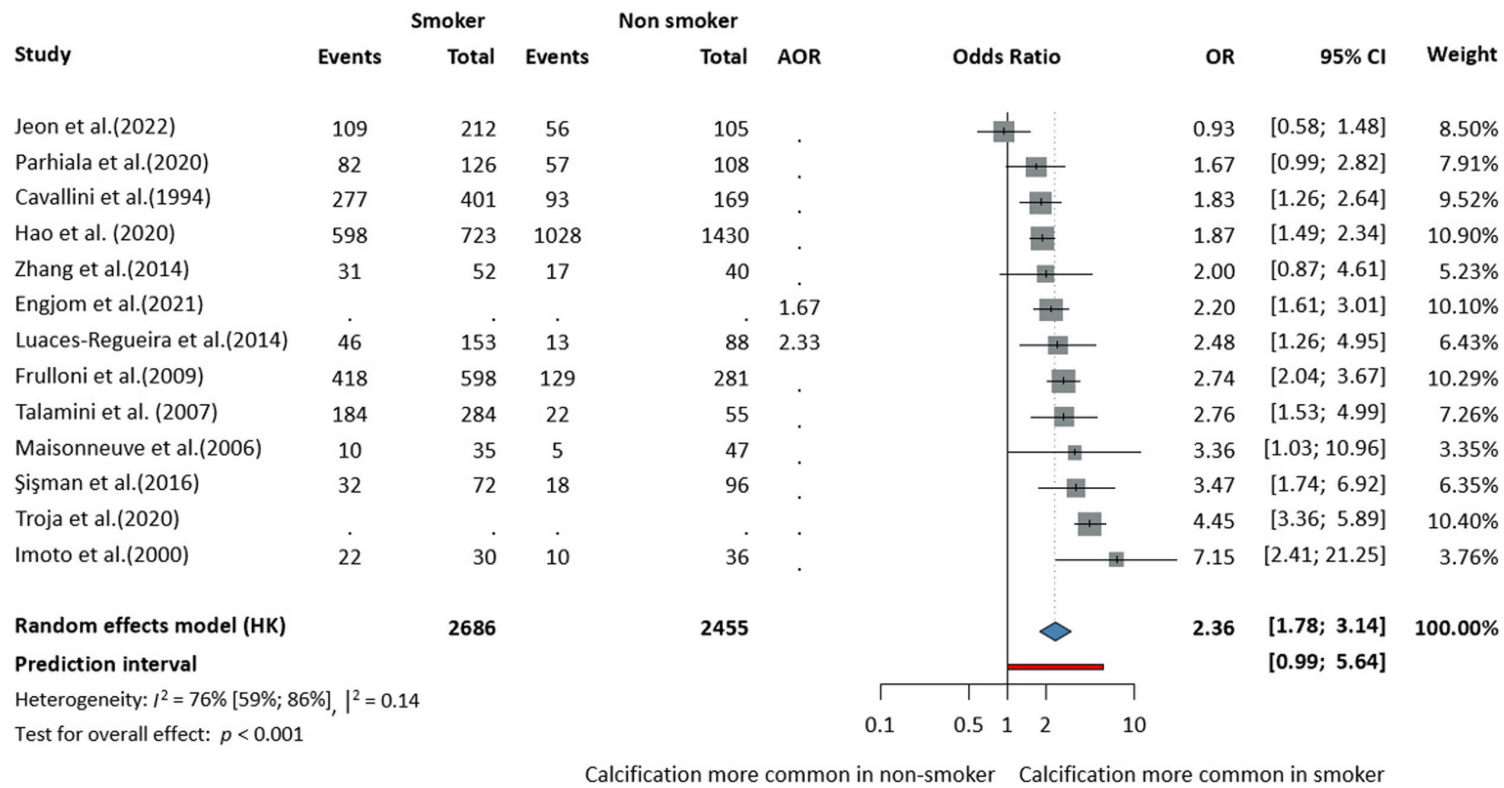
Risk of the calcifying phenotype in chronic pancreatitis in smokers compared to non-smokers (AOR, adjusted odds ratio; CI, confidence interval; OR, odds ratio).

In terms of population characteristics, the CCP phenotype was associated with male sex (OR = 1.27, CI: 1.05–1.53, *I*^2^ = 64% CI: 40–78%), and patients were on average younger at diagnosis compared to those with the non-calcifying phenotype, although this difference was not statistically significant (MD = −2.64, CI:−6.24–0.96, *I*^2^ = 93% CI: 88–96%) (Supplementary Figures S1, S2). This age difference was analyzed across eight studies, including five prospective cohort studies, one retrospective cohort study, and two cross-sectional studies. In terms of lifestyle-related factors, alcohol consumption (OR = 2.31, CI: 1.8–2.98, *I*^2^ = 72% CI: 58–81%, Figure 2) and smoking (OR = 2.22, CI: 1.61–3.06, *I*^2^ = 78% CI: 63–87%, Figure 3) were associated with CCP.

**Figure 4:**
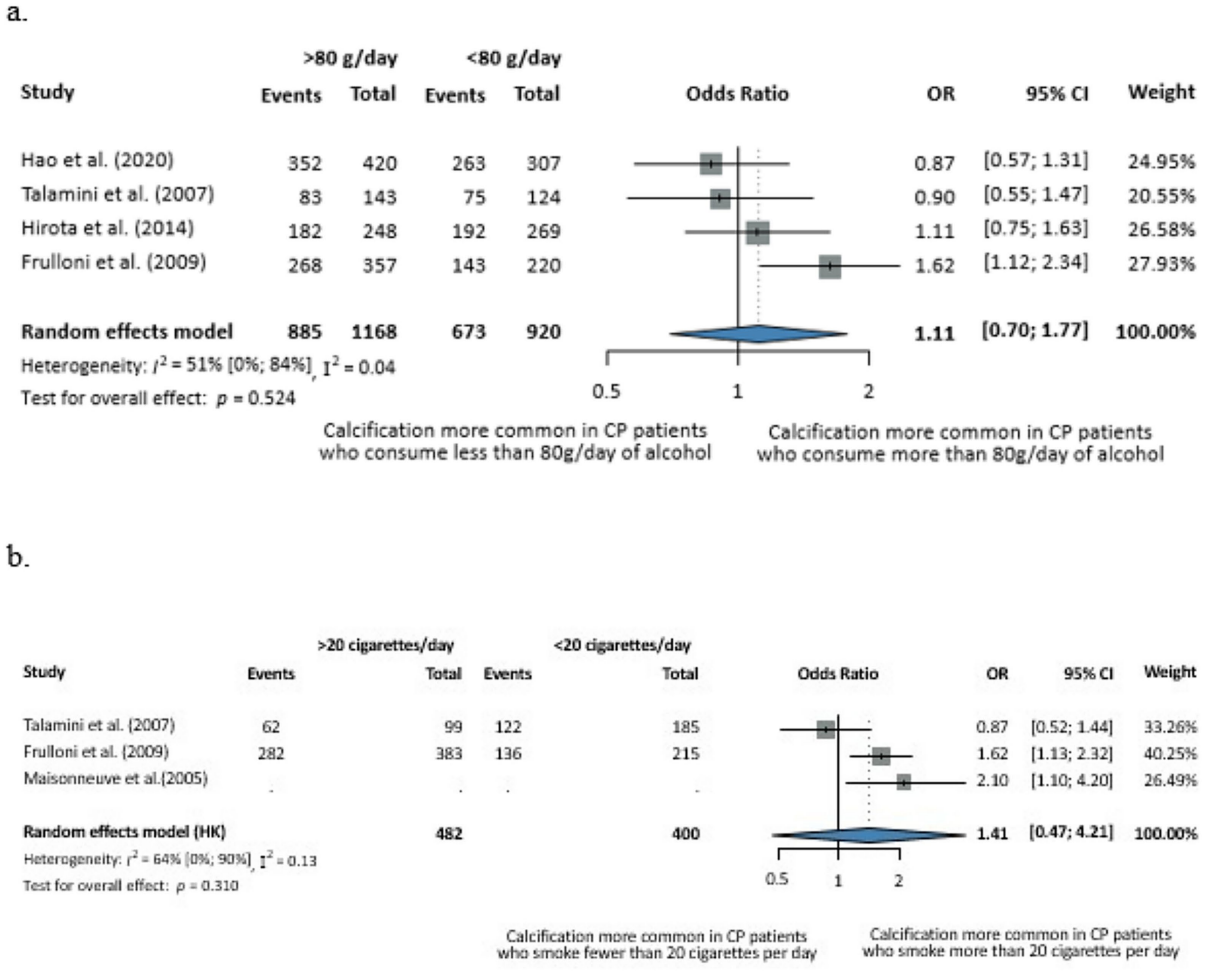
Evaluation of the dose-dependent effects of alcohol consumption **(a)** and smoking **(b)** on calcification in CP. **(a)** Odds of the calcifying phenotype with less versus more than 80 g of alcohol consumption per day. **(b)** Odds of the calcifying phenotype with less versus more than 20 cigarettes smoked per day (CI, confidence interval; CP, chronic pancreatitis; OR, odds ratio).

We further stratified the analysis based on the average amount of daily alcohol consumption and found no statistically significant difference between the groups at a cut-off of 80 g. The number of cigarettes per day made no significant difference either in terms of calcification risk in CP (cut off of 20 cigarettes/day) (Figure 4). However, these subgroup analyses were based on very few studies, with wide confidence intervals, and therefore, the results should be interpreted with caution.

### 3.3 Etiology-related risk factors for calcification in CP

Among the CP etiologies, we investigated alcohol versus hereditary etiology, and there were no differences between the two groups (OR = 1.17, CI: 0.48–2.88, *I*^2^ = 44%, CI:0–78%, Supplementary Figure S3). However, we found a higher odds of calcification among patients with alcoholic compared to idiopathic CP (OR = 1.45, CI: 1.0–2.11, *I*^2^ = 83%, CI: 76–88%, Supplementary Figure S4). Data on genetic factors were scarce. We examined the cystic fibrosis transmembrane conductance regulator (CFTR) mutation and found no significant difference between the groups (OR = 0.43, CI: 0.11–1.66, *I*^2^ = 2% CI: 0–90%, Supplementary Figure S5).

Association of recurrent AP (RAP) made no difference in terms of odds of calcifications overall (OR = 0.76, CI: 0.52–1.11, *I*^2^ = 71% CI: 25–88%, Supplementary Figure S6), or in the subgroup of patients with autoimmune pancreatitis (AIP) (OR = 5.68, CI: 0.59–54.72, *I*^2^ = 71% CI: 0–93%, Supplementary Figure S7). Similar results were obtained when comparing early (before 35 years of age) and late onset CP patients (OR = 1.04, CI: 0.51–2.12, *I*^2^ = 33% CI: 0–73%, Supplementary Figure S8). We also compared the duration of CP between CCP and NCCP patients; on average, it was 1 year longer in CCP patients (MD = 1.01, CI: −0.22-2.24, *I*^2^ = 54% CI: 0–85%, Supplementary Figure S9). This analysis was based on five studies, including one prospective cohort study, two retrospective cohort studies, and two cross-sectional studies. Although follow-up time was not consistently reported across all studies, where available, it ranged from approximately 0 to 20 years.

### 3.4 Risk of bias assessment

Results of the risk of bias assessment for each outcome are presented in the Supplementary Figures S10–S14I. We found that in 70% of the studies, a moderate/high risk of bias was observed for each evaluated outcome, resulting from insufficient reporting on study attrition, suboptimal definitions of outcome measurements, and a moderate risk of selection bias associated with the study population. The assessment of publication bias was performed for alcohol consumption, smoking, sex, and alcohol vs. idiopathic CP, revealingsome risk of small study effect across these analysis. The corresponding funnel plots are presented in the Supplementary Figures S10–S13. We were unable to assess publication bias for the other outcomes due to the small number of available studies.

### 3.5 Heterogeneity

Moderate to substantial heterogeneity was observed in several analyses using the Higgins and Thompson *I*^2^ statistic, which was likely attributable to the varying definitions of CP used across studies, as well as differences in the diagnostic modalities applied to detect calcification. Prediction intervals, shown in the main figures for analyses including seven or more studies, generally aligned with the pooled estimates but highlighted uncertainty in some analyses.

## 4 Discussion

Our results highlight smoking and alcohol consumption as significant modifiable factors associated with pancreatic calcifications in CP, and suggest that alcoholic CP is associated with higher odds of calcification compared to CP with idiopathic etiology. Data on the contribution of genetic factors or age at onset were scarce and heterogeneous, preventing us from drawing strong conclusions. There was no significant difference in disease duration between NCCP and CCP.

Calcifications are common in CP, with up to 90% of patients developing calcifications during long-term follow-up, particularly in alcohol-induced cases (10, 25). The pathogenesis of pancreatic calcification is multifactorial. Alterations in pancreatic ductal bicarbonate secretion play a central role in the precipitation of calcium salts within the ductal system (26). Notably, smoking has been shown to impair bicarbonate secretion, which is essential for maintaining ductal alkalinity and inhibiting intraductal mineral deposition (26). Previous studies have highlighted the complex, synergistic effects of alcohol and smoking in promoting inflammatory and fibrotic changes in the pancreas (10, 27). Consistent with these findings, both smoking and alcohol consumption have been implicated as key risk factors associated with chronic calcific pancreatitis (CCP) (25). There is also a well-established strong correlation between smoking and drinking, suggesting that the effect of smoking may be influenced by alcohol consumption (28). An observational study examining structural changes over time reported that calcification tends to occur earlier in individuals with a history of heavy smoking (29). This accelerated calcification process may be explained by chronic tobacco use contributing to the deposition of calcium salts in the pancreatic ducts (30). Furthermore, population-based evidence from a Danish study underscores smoking as the predominant risk factor for CP, with a greater impact than alcohol, emphasize the central role of smoking in both the initiation and progression of pancreatic calcifications (31).

Although alcohol exhibits both direct toxic effects on pancreatic acinar cells and contributes to ischemic injury, experimental animal models have consistently shown that alcohol alone does not induce pancreatitis (32, 33). It is believed to sensitize the pancreas to other injurious factors, leading to tissue damage through repeated inflammatory episodes (32). In alcoholic pancreatitis, calcification tends to appear earlier than in idiopathic pancreatitis, particularly in the early-onset group (34). Consistently, we found a significantly higher odds of pancreatic calcifications in alcoholic CP compared both to idiopathic CP and to non-alcoholic CP. In contrast, population-based data from the USA indicate that although alcoholic CP generally exhibits a more severe clinical phenothype, there was no significant diffenence in the risk of pancreatic calcification between alcoholic and non-alcoholic CP (35). This supports the proposed role of alcohol in disrupting ductal secretion, as previously discussed, which may create a permissive environment for intraductal calcium precipitation (10). Smoking further exacerbates these processes by activating mechanisms that promote fibrosis (36, 37).

Given these synergistic and compounding effects of alcohol and smoking, lifestyle interventions are particularly important. For individuals with CP, particularly those with genetic predispositions, complete alcohol abstinence is likely to be beneficial (38). Moreover, a previous study suggests that smoking cessation reduces the risk of pancreatic calcifications, highlighting the potential for modifiable risk factors to influence disease progression (39). Although our subgroup analyses did not show a statistically significant dose–response relationship between alcohol consumption or cigarette smoking and calcification risk in CP, the lack of observed associations should not be interpreted as evidence of absence, given the limited number of studies and wide confidence intervals. Consistent with a potential dose-dependent effect, a retrospective Korean study by Lee et al. (30) found that continued smoking significantly accelerated the progression of pancreatic calcification in CP, with a clear dose-dependent association between the amount of smoking and calcification progression. In parallel, a large Chinese study investigating light-to-moderate alcohol consumption-related CP reported that even modest alcohol exposure was associated with a higher prevalence of calcification (38). Similarly, a Scandinavian-Baltic cohort study demonstrated a dose-dependent link between smoking and pancreatic calcifications (40). In addition to lifestyle-related factors, demographic characteristics may also play a role in the risk of pancreatic calcification. As the present analysis is based on unadjusted comparisons, the increased risk of calcification in males may be influenced by confounding factors such as higher prevalence of alcohol consumption and smoking. These behaviors, which are more commonly reported among men, may partially account for the observed sex-related differences in calcification rates, and the association should therefore be interpreted with caution (41, 42).

A previous study found that the duration of CP was found to be positively correlated with the presence of calcifications, changes in the main pancreatic duct (MPD), and a reduction in pancreatic size (pancreatic atrophy) (43). Although age at diagnosis of CP also showed some independent associations, these findings were generally less robust. Consequently, the duration of the disease emerges as the primary time-dependent factor influencing the development of structural changes in CP. Therefore, the time of disease duration should be considered when assessing morphological changes (2).

The relationship between pancreatic calcification and pancreatic dysfunction is debated (44). Recent observational data suggest that the presence of pancreatic calcifications does not correlate with severe exocrine pancreatic insufficiency (45). In contrast, another study suggests a clear association between exocrine insufficiency and pancreatic calcification (44). A previous study showed that the incidence of exocrine insufficiency in alcoholic CP patients increased sharply from approximately 23% before the onset of calcifications to over 90% within two years following the onset of pancreatic calcifications (46). However, the association was much weaker in non-alcoholic CP patients, as in 40% of cases, calcifications appeared up to 13 years or more before the development of exocrine insufficiency (46). In addition to exocrine dysfunction, pancreatic calcifications have been linked to endocrine insufficiency, with studies showing a higher prevalence of diabetes in affected patients, possibly due to *β*-cell dysfunction (47). However, it is unclear whether calcifications directly cause endocrine failure or reflect chronic parenchymal damage, with factors such as islet fibrosis or inflammation potentially playing a role.

Treatment of patients with CP typically focuses on pain relief, diabetes control, and management of steatorrhea (48). As suggested by a previous study, the presence of calcifications predicts a slower resolution of pain (49). Depending on the location and number of stones, management options vary. For a limited number of stones, extracorporeal shock wave lithotripsy (ESWL) followed by ERCP with stone extraction is recommended, particularly for pancreatic head/body stones of at least 5 mm. Endoscopic stone fragmentation and ductal drainage may also be employed. In cases of extensive stone formation, surgical approaches are indicated (50, 51). Proper management of calcifications is essential to improve duct patency, reduce pain, and prevent recurrent episodes, ultimately enhancing long-term disease control (51).

### 4.1 Strengths and limitations

Among the strengths of our study, we highlight that it is the first meta-analysis on this topic, including a large number of studies and participants, and employing a rigorous methodology. Notably, although AIP is a chronic condition, the five studies focusing on AIP were evaluated separately from those involving typical CP populations to maintain consistency across analyses.

However, we must also acknowledge the limitations of our study. A moderate to high degree of heterogeneity was observed in several analyses, likely attributable to the use of different diagnostic criteria for chronic CP and the application of varied imaging modalities for detecting calcifications across the included studies. The imagine modalities differ in their sensitivity for identifying pancreatic calcifications, which may contribute to methodological heterogeneity and should be considered when interpreting the pooled estimates. Prediction intervals, shown in the main figures for analyses including seven or more studies, were generally consistent with the pooled estimates but highlighted uncertainty in some analyses. Additionally, for some of the outcomes assessed, the risk of bias was rated as moderate to high, and there was a lack of adjusted odds ratios. Furthermore, the disease duration of CP was not reported in most of the included studies, limiting the possibility of accounting for disease progression as a potential confounding factor. Finally, the subgroup analysis assessing alcohol and smoking exposure categories was based on a limited number of studies, therefore, the results should be interpreted cautiously as exploratory and hypothesis-generating rather than confirmatory.

### 4.2 Implications for practice and research

Given that alcohol consumption and smoking are significantly associated with the risk for calcification in CP, it is crucial to identify these factors in affected patients and incorporate smoking cessation and alcohol reduction programs as part of the standard of care consistent with current international guidelines (48). Our results further underscore this established clinical priority by demonstrating the association between these modifiable behaviors and the calcifying CP phenotype. Recent clinical data indicate that alcohol cessation substantially reduces the risk of pancreatic calcification, whereas cumulative tobacco exposure remains a key determinant of calcification severity (52). Addressing these modifiable behaviors may help prevent or mitigate the progression of calcification and its associated complications, ultimately improving patient outcomes (53, 54).

Further research is needed to better understand the underlying mechanisms the observed associations between alcohol consumption, smoking, and the development of calcification in CP. Moreover, the pathophysiology and dynamics of pancreatic calcifications need further exploration. Future well-designed, prospective studies are required to confirm these associations and to reduce the uncertainty caused by bias and confounding in the current evidence base.

## 5 Conclusion

Modifiable risk factors as alcohol consumption and smoking were significantly associated with calcifying CP phenotype. It is essential to incorporate smoking cessation and alcohol consumption reduction programs into CP standard of care.

## Data Availability

The original contributions presented in the study are included in the article/Supplementary material, further inquiries can be directed to the corresponding author.
