# Correction: Risk factors for calcification in chronic pancreatitis: a systematic review and meta-analysis

**DOI:** 10.3389/fmed.2026.1792540

**Published:** 2026-03-13

**Authors:** Orsolya Eperjesi, Jázmin Németh, Anett Rancz, Karen Krisztina Fazekas, Brigitta Teutsch, Renáta Papp, Peter Hegyi, Stefania Bunduc

**Affiliations:** 1 Centre for Translational Medicine, Semmelweis University, Budapest, Hungary; 2 Department of Internal Medicine, Toldy Ferenc Hospital, Cegléd, Hungary; 3 Institute of Pancreatic Diseases, Semmelweis University, Budapest, Hungary; 4 Institute for Translational Medicine, Medical School, University of Pécs, Pécs, Hungary; 5 Department of Radiology, Medical Imaging Centre, Semmelweis University, Budapest, Hungary; 6 Department of Pharmacology and Pharmacotherapy, Semmelweis University, Budapest, Hungary; 7 Center for Pharmacology and Drug Research & Development, Semmelweis University, Budapest, Hungary; 8 Centre of Science and Innovation Vice-rector and Business Development, Semmelweis University, Budapest, Hungary; 9 Translational Pancreatology Research Group, Interdisciplinary Centre of Excellence for Research Development and Innovation, University of Szeged, Szeged, Hungary; 10 Faculty of Medicine, Carol Davila University of Medicine and Pharmacy Bucharest, Bucharest, Romania; 11 Digestive Disease and Liver Transplant Center, Fundeni Clinical Institute, Bucharest, Romania

**Keywords:** alcohol drinking, pancreatic calculi, smoking, tobacco use disorder, alcohol-related disorders

Author “Brigitta Teutsch” was erroneously spelled as “Teutsch Brigitta.”

Author “Stefania Bunduc” was erroneously assigned to affiliation “Carol Davila University of Medicine and Pharmacy, Bucharest, Romania”. This affiliation has now been removed for author “Stefania Bunduc.” The affiliation “Faculty of Medicine, Carol Davila University of Medicine and Pharmacy Bucharest, Bucharest, Romania” is now listed first for Stefania Bunduc.”

There was a mistake in Figure 3 as published. The data for the study by Talamini et al. (2007) were incorrectly displayed in the original figure. The corrected Figure 3 appears below.

**Figure 3:**
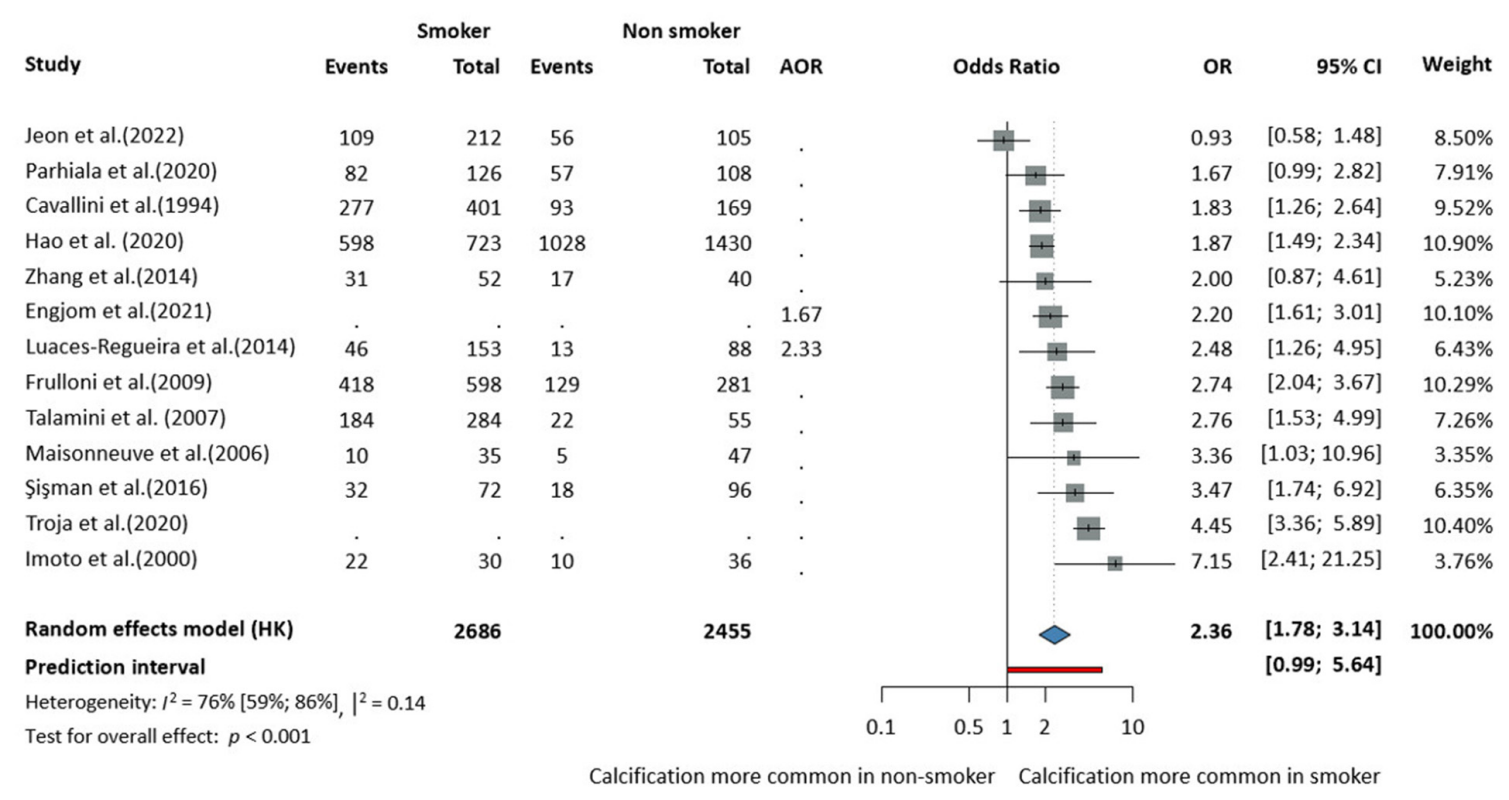
Risk of the calcifying phenotype in chronic pancreatitis in smokers compared to non-smokers (AOR, adjusted odds ratio; CI, confidence interval; OR, odds ratio).

In the published article, the manuscript was incorrectly classified as “Original Research.” The correct article type is “Systematic Review.”

The original version of this article has been updated.

## Publisher's note

All claims expressed in this article are solely those of the authors and do not necessarily represent those of their affiliated organizations, or those of the publisher, the editors and the reviewers. Any product that may be evaluated in this article, or claim that may be made by its manufacturer, is not guaranteed or endorsed by the publisher.

