# Supplementary material for: Risk factors for calcification in chronic pancreatitis: a systematic review and meta-analysis
Source: Front Med (Lausanne). 2025 Nov 19;12:1703088. doi: 10.3389/fmed.2025.1703088 (PMC12673887; doi:10.3389/fmed.2025.1703088)
Supplement: Supplementary file 1 [file Data_Sheet_1.pdf]

SUPPLEMENTARY MATERIAL

SUPPLEMENTARY MATERIALS

METHODS

Table S1. PRISMA checklist

RESULTS

Table S2. Basic characteristics of studies included

Table S3. Qualitative analysis

Figure S1-9 Forest plots

Figure S10-13 Publication bias assessment

Figure S14A-I Risk of bias assessment

REFERENCE

Table S1. PRISMA checklist

Section and Topic	Item #	Checklist item	Location where item is reported
**TITLE**			
Title	1	Identify the report as a systematic review.	p.1
**ABSTRACT**			
Abstract	2	See the PRISMA 2020 for Abstracts checklist.	p.2
**INTRODUCTION**			
Rationale	3	Describe the rationale for the review in the context of existing knowledge.	p.3
Objectives	4	Provide an explicit statement of the objective(s) or question(s) the review addresses.	p.3
**METHODS**			
Eligibility criteria	5	Specify the inclusion and exclusion criteria for the review and how studies were grouped for the syntheses.	p.4
Information sources	6	Specify all databases, registers, websites, organisations, reference lists and other sources searched or consulted to identify studies. Specify the date when each source was last searched or consulted.	p.3
Search strategy	7	Present the full search strategies for all databases, registers and websites, including any filters and limits used.	p.4
Selection process	8	Specify the methods used to decide whether a study met the inclusion criteria of the review, including how many reviewers screened each record and each report retrieved, whether they worked independently, and if applicable, details of automation tools used in the process.	p.4
Data collection process	9	Specify the methods used to collect data from reports, including how many reviewers collected data from each report, whether they worked independently, any processes for obtaining or confirming data from study investigators, and if applicable, details of automation tools used in the process.	p.4
Data items	10a	List and define all outcomes for which data were sought. Specify whether all results that were compatible with each outcome domain in each study were sought (e.g. for all measures, time points, analyses), and if not, the methods used to decide which results to collect.	p.4-5
	10b	List and define all other variables for which data were sought (e.g. participant and intervention characteristics, funding sources). Describe any assumptions made about any missing or unclear information.	p.4-5
Study risk of bias assessment	11	Specify the methods used to assess risk of bias in the included studies, including details of the tool(s) used, how many reviewers assessed each study and whether they worked independently, and if applicable, details of automation tools used in the process.	p.5

Section and Topic	Item #	Checklist item	Location where item is reported
Effect measures	12	Specify for each outcome the effect measure(s) (e.g. risk ratio, mean difference) used in the synthesis or presentation of results.	p.4-5
Synthesis methods	13a	Describe the processes used to decide which studies were eligible for each synthesis (e.g. tabulating the study intervention characteristics and comparing against the planned groups for each synthesis (item #5)).	p.4-5
	13b	Describe any methods required to prepare the data for presentation or synthesis, such as handling of missing summary statistics, or data conversions.	p.4-5
	13c	Describe any methods used to tabulate or visually display results of individual studies and syntheses.	p.4-5
	13d	Describe any methods used to synthesize results and provide a rationale for the choice(s). If meta-analysis was performed, describe the model(s), method(s) to identify the presence and extent of statistical heterogeneity, and software package(s) used.	p.4-5
	13e	Describe any methods used to explore possible causes of heterogeneity among study results (e.g. subgroup analysis, meta-regression).	p.4-5
	13f	Describe any sensitivity analyses conducted to assess robustness of the synthesized results.	p.4-5
Reporting bias assessment	14	Describe any methods used to assess risk of bias due to missing results in a synthesis (arising from reporting biases).	p.5
Certainty assessment	15	Describe any methods used to assess certainty (or confidence) in the body of evidence for an outcome.	NA
**RESULTS**			
Study selection	16a	Describe the results of the search and selection process, from the number of records identified in the search to the number of studies included in the review, ideally using a flow diagram.	p.5
	16b	Cite studies that might appear to meet the inclusion criteria, but which were excluded, and explain why they were excluded.	NA
Study characteristics	17	Cite each included study and present its characteristics.	Suppl.p.6-12
Risk of bias in studies	18	Present assessments of risk of bias for each included study.	p.7, Suppl.p.21-30
Results of individual studies	19	For all outcomes, present, for each study: (a) summary statistics for each group (where appropriate) and (b) an effect estimate and its precision (e.g. confidence/credible interval), ideally using structured tables or plots.	NA
Results of syntheses	20a	For each synthesis, briefly summarise the characteristics and risk of bias among contributing studies.	p.5-7

Section and Topic	Item #	Checklist item	Location where item is reported
	20b	Present results of all statistical syntheses conducted. If meta-analysis was done, present for each the summary estimate and its precision (e.g. confidence/credible interval) and measures of statistical heterogeneity. If comparing groups, describe the direction of the effect.	p.5-7, Suppl.p.15-20
	20c	Present results of all investigations of possible causes of heterogeneity among study results.	p.7
	20d	Present results of all sensitivity analyses conducted to assess the robustness of the synthesized results.	NA
Reporting biases	21	Present assessments of risk of bias due to missing results (arising from reporting biases) for each synthesis assessed.	p.7, Suppl.p.21-24
Certainty of evidence	22	Present assessments of certainty (or confidence) in the body of evidence for each outcome assessed.	NA
**DISCUSSION**			
Discussion	23a	Provide a general interpretation of the results in the context of other evidence.	p.7
	23b	Discuss any limitations of the evidence included in the review.	p.9-10
	23c	Discuss any limitations of the review processes used.	p.9-10
	23d	Discuss implications of the results for practice, policy, and future research.	p.10
**OTHER INFORMATION**			
Registration and protocol	24a	Provide registration information for the review, including register name and registration number, or state that the review was not registered.	p.3
	24b	Indicate where the review protocol can be accessed, or state that a protocol was not prepared.	p.3
	24c	Describe and explain any amendments to information provided at registration or in the protocol.	p.3
Support	25	Describe sources of financial or non-financial support for the review, and the role of the funders or sponsors in the review.	p.10
Competing interests	26	Declare any competing interests of review authors.	p.11
Availability of data, code and other materials	27	Report which of the following are publicly available and where they can be found: template data collection forms; data extracted from included studies; data used for all analyses; analytic code; any other materials used in the review.	NA

TABLE S2. BASIC CHARACTERISTICS OF STUDIES INCLUDED

Study (year)	Study design	Study period	Follow-up months (range)	Country	Disease duration (months)	Nr. of all patients with CP	Frequency of patients with calcification (%)	Diagnosis of calcification	Sex Male %	Age-Mean±SD (median±range)	Frequency of smokers (%)	Frequency of drinkers (%)
Agarwal et al.(2020)[1]	prospective cohort study	1998-2019	NR	India	NR	1415	80.4	NR	78	34±12	12	38
Amman et al.(1987)[2]	prospective, observational cohort study	1963-1986	24-312	Switzerland	NR	287	85.4	x-ray	88	*46(16-78)*	NR	71
Amman et al.(2007)[3]	prospective, observational cohort study	NR	NR	Switzerland	NR	343	85	NR	87	NR	NR	77
Ammann et al.(1994)[4]	prospective, observational cohort study	1963-1992	0-300	Switzerland	NR	254	12	x-ray	NR	NR	NR	NR
Arvanitakis et al. (2004)[5]	prospective observational study	1995-2000	NR	Belgium	69.8±5.4	146	74.7	CT	77.4	50±1	NR	74.4
Anderson et al.(1988)[6]	case-controll study	NR	NR	UK	NR	88	44	NR	76	NR	NR	59
Angelis et al.(1992)[7]	retrospective, comparative histopathological study	1981-1987	NR	Italy	NR	42	43	NR	NR	NR	60	62
Bhadada et al.(2007)[8]	retrospective, observational, cross-sectional study	2001-2005	NR	India	NR	27	55	NR	59	NR	NR	33
Bhasin et al.(2011)[9]	cross-sectional study	1999-2009	NR	India	NR	225	46.7	x-ray, US, CT	79.1	36.2±12.5	43	NR
Buscail et al.(1995)[10]	prospective cohort study	1990-1992	6-24	France	NR	44	55	NR	89	NR	NR	100
Casals et al.(2004)[11]	prospective cohort study	2000-2002	12-300	Spain	NR	68	63	x-ray, US, CT	79	NR	52	54

CT - Computed Tomography; CP - Chronic Pancreatitis; EUS - Endoscopic Ultrasound; ERCP - Endoscopic Retrograde Cholangiopancreatography; MRCP - Magnetic Resonance Cholangiopancreatography; SD - Standard Deviation; NR - Not Reported; US - Ultrasound

TABLE S2. BASIC CHARACTERISTICS OF STUDIES INCLUDED

Cavallini et al.(1994)[12]	prospective cohort study	1973-1989	NR	Italy	NR	570	66	x-ray, US, CT, ERCP,	NR	NR	NR	88
Cavallini et al.(1998)[13]	prospective cohort study	1971-1995	12-300	Italy	NR	715	76	US/CT	88	41±11	88	75
Chari et al.(1992)[14]	retrospective study	1987-1989	NR	India	NR	209	87	X-ray and US	NR	NR	NR	24
Coté et al.(2011)[15]	case control study	2000-2006	NR	US	NR	539	55	CT/ERCP	53	49.5±15	71	44
Dani et al.(1986)[16]	retrospective cohort study	1963-1986	NR	Brazil	NR	329	81	x-ray, histology	91	NR	NR	86
Dítě et al. (2012)[17]	prospective cohort study	1992-2003	NR	Czech Republic, United Arab Emirates	NR	223	5	NR	66,4	NR	63	73
Ectors et al. (1997)[18]	retrospective study	NR	NR	Germany, Belgium	NR	24	75	NR	70	NR	NR	50
Eloubeidi et al.(2013)[19]	prospective cohort study	2008-2009	NR	USA	NR	164	10	NR	37	NR	NR	NR
Engjom et al.(2021)[20]	prospective, cross-sectional study	2016-2019	NR	Norway	*60(12-120)*	959	65	US/CT/EUS	66	55±14	81	60
Frulloni et al.(2008)[21]	prospective, cross-sectional study	2001-2006	NR	Italy	NR	198	76	x-ray and/or CT	65	40±14.9	NR	NR
Frulloni et al.(2009)[22]	prospective, cross-sectional study	2000-2005	NR	Italy	NR	893	62	x-ray and/or CT	74	53.7±15.2	66	60
Gasiorowska et al.(2011)[23]	cross-sectional observational study	NR	NR	Poland	NR	47	NR	US,CT	74	NR	NR	70
Hao et al.(2020)[24]	retrospective-prospective cohort	2000-2013	0-638.4	China	NR	2153	75.5	CT,ERCP,MRI,EUS	69	38±17	34	34
Hayakawa et al.(1995)[25]	cross-sectional study	NR	NR	Japan	NR	19	32	NR	89	57±2.9 (28-77)	NR	NR

CT - Computed Tomography; CP - Chronic Pancreatitis; EUS - Endoscopic Ultrasound; ERCP - Endoscopic Retrograde Cholangiopancreatography; MRCP - Magnetic Resonance Cholangiopancreatography; SD - Standard Deviation; NR - Not Reported; US - Ultrasound

TABLE S2. BASIC CHARACTERISTICS OF STUDIES INCLUDED

Hayakawa et. al.(1989)[26]	prospective cohort study	NR	NR	Japan	NR	155	49	NR	82	NR	NR	57
Hirota et al.(2014)[27]	cross-sectional study	2011(January-december)	NR	Japan	NR	1518	70	US,CT,MRCP,EUS,ERCP	NR	NR	75	NR
Hirth et al. (2018)[28]	retrospective, cross-sectional	1998-2007	2-84	Germany, Ukraine	29	741	8	NR	63	NR	8	13
Imoto et al.(2000)[29]	prospective cohort study	1976-1985	NR	USA	NR	66	48	NR	52	NR	45	NR
James et al.(1974)[30]	retrospective cohort study	1968-1973	NR	UK	NR	107	28	NR	67	NR	NR	42
Jeon et al.(2022)[31]	prospective cohort study	2008-2014	NR	USA	NR	317	52	CT/MRI/MRCP/EUS	59	*53(45-59)*	85	22
Kamisawa et al.(2004)[32]	retrospective study	1976-2003	NR	Japan	NR	182	45	NR	89	57.7±8.2	NR	74
Kanai et al.(2016)[33]	retrospective cohort study	1992-2014	36-301	Japan	NR	92	18	NR	77	*74(55-90)*	NR	NR
Kawa et al.(2002)[34]	retrospective cohort study	1994-2001	NR	Japan	NR	83	52	NR	84	NR	NR	NR
Kawa et al. (2009)[35]	prospective observational study	NR	NR	Japan	NR	51	17.6	ERCP/MRCP	NR	NR	NR	NR
Keim et al. (2003)[36]	case-control-study	NR	NR	Germany	NR	550	NR	NR	NR	NR	NR	NR
Kuraishi et al.(2020)[37]	retrospective study	1996-2018	14-284	Japan	NR	145	NR	CT	74	*67(38-92)*	57	45
Lankisch et al.(1986)[38]	cross-sectional study	NR	NR	Germany	NR	79	70	x-ray	NR	NR	NR	65
Lankisch et al.(2001)[39]	retrospective and prospective cohort study	1976-1985	NR	Germany	NR	372	54	NR	65	NR	NR	82
Layer et al.(1994)[40]	retrospective cohort study	1976-1982	NR	US	NR	315	56	NR	68	NR	NR	79

CT - Computed Tomography; CP - Chronic Pancreatitis; EUS - Endoscopic Ultrasound; ERCP - Endoscopic Retrograde Cholangiopancreatography; MRCP - Magnetic Resonance Cholangiopancreatography; SD - Standard Deviation; NR - Not Reported; US - Ultrasound

TABLE S2. BASIC CHARACTERISTICS OF STUDIES INCLUDED

	with prospective follow-up											
Law et al.(2010)[41]	retrospective, cross-sectional study	2006-2008	NR	USA	NR	79	56	CT/EUS	53	NR	62	NR
Lee et al.(2016)[42]	retrospective study	2002-2010	17.1-112.7	Korea	NR	59	63	CT	90	54±15	80	85
Lewis et al.(2020)[43]	cross-sectional study	2000-2014	NR	USA	NR	663	55	EUS	55	NR	41	83
Luaces-Regueira et al.(2014)[44]	prospective, cross-sectional case-case study	2006-2010	NR	Spain	NR	241	24.5	EUS	30742	45.2±14.4	63	67
Machicado et al.(2017)[45]	retrospective cohort study	1977-2006	0.1-29.4	USA	NR	89	70	NR	56	*56(48-67)*	75	52
Maisonneuve et al.(2005)[46]	retrospective cohort study	NR	NR	Italy	NR	934	40	NR	87	45	93	72
Maisonneuve et al.(2006)[47]	retrospective cohort study	NR	NR	Italy, Switzerland	NR	166	64	US/CT	73	NR	60	21
Maruyama et al. (2011)[48]	prospective observational study	NR	36-230	Japan	NR	69	25	CT	NR	NR	NR	NR
Midha et al.(2010)[49]	prospective observational + case-control study	2004-2008	NR	India	110.16±70.08	411	75	NR	28216	29.83±12.44	NR	38
Miyake et al.(1989)[50]	prospective cohort study	NR	12-252	Japan	NR	135	55	x-ray and/or CT	76	51.8±13.3	69	62
Müllhaupt et al. (2005)[51]	prospective cohort study	NR	NR	Switzerland	NR	343	85	NR	81	NR	NR	NR
Nakamura et al.(1996)[52]	prospective cohort study	NR	NR	Japan	NR	85	22	x-ray/CT/ERCP	NR	NR	NR	NR
Nojgaard et al.(2010)[53]	prospective cohort study	1977-1982	NR	Denmark	NR	290	26	NR	72	NR	NR	44

CT - Computed Tomography; CP - Chronic Pancreatitis; EUS - Endoscopic Ultrasound; ERCP - Endoscopic Retrograde Cholangiopancreatography; MRCP - Magnetic Resonance Cholangiopancreatography; SD - Standard Deviation; NR - Not Reported; US - Ultrasound

TABLE S2. BASIC CHARACTERISTICS OF STUDIES INCLUDED

Oh et al.(2017)[54]	prospective cohort study	2012-2015	NR	USA	NR	211	53	X-ray/CT/ERCP.	42	NR	4	39
Olesen et al.(2019)[55]	prospective cross-sectional study	2016-2019	NR	Scandinavian-Baltic region+Russia	51.6±66	1509	60	CT, MRCP, transabdominal and endoscopic US	67	53.9±14.5	59	56
Paolini et al.(1998)[56]	prospective cohort study	1973-1996	12-276	France	NR	134	57	NR	89	NR	NR	91
Parhiala et al.(2020)[57]	retrospective cohort study	2014-2015	NR	Finland	*48(12-504)*	235	66	NR	65	*57(26-88)*	54	68
Rajesh et al.(2014)[58]	retrospective+prospective study	2004-2010	NR	India	NR	597	33	US/CT/ERCP/MRCP/EUS	NR	NR	NR	29
Rebours et al.(2012)[59]	prospective observational study	2006-2009	NR	France	NR	108	71	CT/EUS	88	*45(26-69)*	NR	NR
Regunath et al.(2010)[60]	prospective observational study	2007-2009	NR	India	*13(1-30)*	54	51.1	X-ray, US, CT, ERCP/MRCP	87	34.5±12	NR	28
Robles-Díaz et al.(1990)[61]	retrospective cohort study	1975-1980, 1982-1987	NR	Mexico	NR	150	74	NR	NR	NR	NR	67
Romagnuolo et al.(2016)[62]	prospective cohort study	2008-2011	NR	US	NR	521	55	EUS	55	*45(31-55)*	56	53
Ru et al.(2021)[63]	cross-sectional study	2011-2015	NR	China	NR	1022	72.3	NR	70	*37.5(25-48)*	47	47
Sarles et al.(1979)[64]	cross-sectional study	NR	NR	19 countries	NR	1615	58	x-ray	82	NR	NR	NR
Schnelldorfer et al.(2008)[65]	retrospective cohort study	1995-2003	NR	US	NR	372	31	NR	52	*46(14-74)*	NR	46

CT - Computed Tomography; CP - Chronic Pancreatitis; EUS - Endoscopic Ultrasound; ERCP - Endoscopic Retrograde Cholangiopancreatography; MRCP - Magnetic Resonance Cholangiopancreatography; SD - Standard Deviation; NR - Not Reported; US - Ultrasound

TABLE S2. BASIC CHARACTERISTICS OF STUDIES INCLUDED

Shalimar et al.(2017)[66]	prospective observational study	2008-2011	NR	India	106.44±74.88	313	77	US, ERCP, MRCP	78	26.2±12.2	28	26
Sinha et al. (2016)[67]	retrospective cohort study	2003-2013	NR	USA	NR	60	60	NR	53	51.6±12.5	50	40
Singh et al.(2023)[68]	prospective observational study	NR	NR	India	NR	100	53	NR	77	NR	NR	49
Şişman et al.(2016)[69]	retrospective cohort study	2007-2013	6-55	Turkey	59 mean(12–120)	168	30	CT	81	46±15.2	8	39
Takayama et al. (2004)[70]	prospective cohort study	1994-2002	13-111	Japan	NR	42	19	NR	86	*64(38±76)*	NR	N
Talamini et al.(2007)[71]	prospective cohort study	NR	NR	Italy	NR	360	59.8	X-ray/US/CT	32143	14062	88	72
Tao et al.(2022)[72]	retrospective observational study	2012-2020	3-23	China	NR	274	32	CT, MRI, ERCP	74	48.63±16	NR	35
Thulivat et al.(2003)[73]	retrospective cohort study	1979-1989	NR	US	NR	193	41	x-ray/ CT	65	14277	NR	66
Tjora et al.(2020)[74]	retrospective cohort study	NR	NR	7 countries	NR	932	59	NR	63	55.5±20	69	32
Truninger et al.(2002)[75]	retrospective cohort study	January 1-decembe r 31, 1998	NR	Switzerland	NR	73	70	NR	88	NR	NR	67
Wang et al. (2009)[76]	retrospective cohort study	2000-2007	NR	China	NR	180	42	NR	79	NR	NR	NR
Wang et al.(2023)[77]	cross-sectional study	2010-2015	NR	China	NR	1061	84	CT/MRI/EUS	70	NR	37	19
Wilcox et al.(2016)[78]	prospective cohort study	2000-2014	NR	USA	NR	1159	57	CT, MRCP, ERCP, EUS, hystology	55	NR	75	52

CT - Computed Tomography; CP - Chronic Pancreatitis; EUS - Endoscopic Ultrasound; ERCP - Endoscopic Retrograde Cholangiopancreatography; MRCP - Magnetic Resonance Cholangiopancreatography; SD - Standard Deviation; NR - Not Reported; US - Ultrasound

TABLE S2. BASIC CHARACTERISTICS OF STUDIES INCLUDED

Zhang et al.(2014)[79]	retrospective cohort study	1999-2013	NR	China	54+39.6	195	42	US, CT, MRCP, ERCP, EUS	68	49±9.5	62	48
Zou et al. (2018)[80]	case-control-study	2010-2015	NR	China	NR	1061	84	NR	70	NR	13	19

CT - Computed Tomography; CP - Chronic Pancreatitis; EUS - Endoscopic Ultrasound; ERCP - Endoscopic Retrograde Cholangiopancreatography; MRCP - Magnetic Resonance Cholangiopancreatography; SD - Standard Deviation; NR - Not Reported; US - Ultrasound

Table S3 Qualitative analysis

**CP etiology and genetic factors as a risk of Calcification**<br><br>Alcoholic chronic pancreatitis (ACP) has been consistently associated with a higher prevalence of pancreatic calcification. Ectors et al. (1997) reported that calcifications were present in 75% of patients with ACP, while none were observed in the non-alcoholic CP group.[18] Similarly, Chari et al. (1992) found comparable calcification frequencies in ACP (90%) and tropical pancreatitis (TCP) (84%), although the radiological features differed—TCP stones were larger, dense, and well-marginated, whereas ACP calculi were small and speckled.[14]<br><br>Toxic CP patients, as reported by Sinha et al. (2016), showed a higher incidence of calcification compared to those with idiopathic CP. Genetic forms of CP also influence calcification tendencies: Zou et al. (2018) identified increased risk of pancreatic stones in patients with SPINK1, PRSS1, CTRC, and CFTR mutations, and Keim et al. (2003) reported significantly more calcifications in SPINK1 compared to PRSS1 mutation carriers.[36, 80]
**Smoking and Pancreatic Calcification**<br><br>Smoking has emerged as a notable factor in the development and progression of pancreatic calcification. Rebours et al. (2012) observed a higher frequency of calcifications among patients with over 20 pack-years of smoking (71%) compared to those with lower exposure (50%).[59] Lee et al. (2016) also found accelerated calcification progression among smokers.[42]
**Autoimmune pancreatitis and calcification**<br><br>Autoimmune pancreatitis (AIP) is typically associated with a lower incidence of calcification. Kawa et al. (2002) found distinct HLA patterns in AIP patients compared to those with calcifying chronic pancreatitis (CCP).[34] Furthermore, Kuraishi et al. (2020) noted that patients receiving corticosteroid therapy had significantly reduced rates of pancreatic calcification, implying a protective or modifying role of immunosuppressive treatment in disease progression.[37]

**Age at CP onset**<br><br>Findings on age-related differences in calcification are mixed. Hirth et al. (2018) found no significant difference in calcification prevalence between elderly (≥60 years) and younger CP patients (30% vs. 31%), suggesting that calcification is a general marker of disease progression.[28]. Conversely, Kamisawa et al. (2004) reported significantly lower calcification rates in patients with late-onset CP (≥65 years), indicating a potential link between age at onset and disease phenotype, with elderly-onset cases tending toward a milder, less calcified course.[32]
**Other Observations**<br><br>Arvanitakis et al. (2004) concluded that K-ras mutations did not influence the presence of calcifications, while Wang et al. (2009) observed no difference in calcification between patients with and without eosinophilia, indicating that eosinophilic involvement may not influence stone formation.[5, 76]

Conclusion

Overall, the presence and progression of pancreatic calcification appear to be influenced by a complex interplay of etiological, environmental, genetic, and demographic factors.

Figure S1 Odds of calcification in chronic pancreatitis among males compared to females

Study	Male Events	Male Total	Female Events	Female Total	AOR	OR	95% CI	Weight
Hayakawa et al.(1995)	5	17	1	2		0.42	[0.02; 8.05]	0.35%
James et al.(1974)	18	72	12	35		0.64	[0.27; 1.54]	3.07%
Cavallini et al.(1998)	476	630	68	85		0.77	[0.44; 1.36]	5.56%
Amman et al.(1987)	209	253	29	34		0.82	[0.30; 2.23]	2.49%
Ru et al.(2021)	511	717	228	305		0.84	[0.62; 1.14]	9.23%
Hao et al.(2020)	1124	1486	502	667		1.02	[0.83; 1.26]	10.75%
Talamini et al.(2007)	187	317	25	43		1.04	[0.54; 1.98]	4.71%
Luaces-Regueira et al.(2014)	.	.	.	.	1.95	1.06	[0.47; 2.39]	3.44%
Olesen et al.(2019)	622	1006	290	503		1.19	[0.96; 1.48]	10.66%
Maisonneuve et al.(2005)	326	814	41	120		1.29	[0.86; 1.92]	7.66%
Şişman et al.(2016)	42	136	8	32		1.34	[0.56; 3.23]	3.07%
Oh et al.(2017)	51	89	61	122		1.34	[0.77; 2.33]	5.70%
Anderson et al.(1988)	31	67	8	21		1.40	[0.51; 3.82]	2.49%
Romagnuolo et al.(2016)	174	287	113	234		1.65	[1.16; 2.34]	8.50%
Sarles et al.(2009)	801	1326	134	289		1.76	[1.37; 2.28]	10.04%
Buscail et al.(1995)	22	39	2	5		1.94	[0.29; 12.95]	0.81%
Frulloni et al.(2009)	449	660	109	232		2.40	[1.77; 3.26]	9.22%
Eloubeidi et al.(2013)	10	60	6	104		3.27	[1.12; 9.50]	2.25%
**Random effects model (HK)**		**7976**		**2833**		**1.27**	**[1.05; 1.53]**	**100.00%**
**Prediction interval**							**[0.72; 2.24]**	

Heterogeneity: I^2 = 64% [40%; 78%], |^2 = 0.06

Test for overall effect: p = 0.017

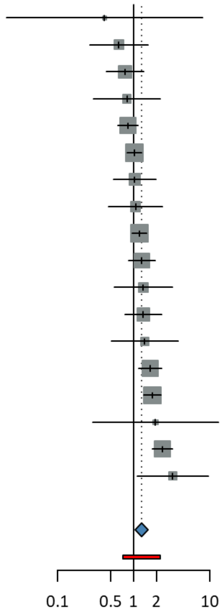

CI: confidence interval, OR: odds ratio, AOR: adjusted odds ratio

Figure S2 Mean differences (MD) in age between patients with calcifying chronic pancreatitis (CCP) and non-calcifying chronic pancreatitis (NCCP)

Study	Age of CCP patients N	Mean	SD	Age of NCCP patients N	Mean	SD	MD	95% CI	Weight
Eloubeidi et al.(2013)	16	50.40	10.50	148	58.40	14.70	-8.00	[-13.66; -2.34]	10.75%
Hao et al. (2020)	1626	41.20	15.40	527	48.89	14.54	-7.70	[-9.15; -6.25]	16.06%
Hayakawa et al.(1995)	6	54.00	7.40	13	59.00	2.60	-5.00	[-11.09; 1.09]	10.19%
Nakamura et al.(1996)	19	50.10	8.70	14	54.90	8.90	-4.80	[-10.89; 1.29]	10.19%
Ammann et al.(1994)	185	38.37	10.05	30	40.33	9.97	-1.96	[-5.81; 1.89]	13.28%
Buscail et al.(1995)	24	48.00	9.00	20	47.00	10.00	1.00	[-4.67; 6.67]	10.74%
Şişman et al.(2016)	50	48.80	10.90	118	46.80	16.20	2.00	[-2.20; 6.20]	12.78%
Olesen et al.(2019)	912	54.30	13.80	597	52.10	15.30	2.20	[0.68; 3.72]	16.00%
**Random effects model**	**2838**			**1467**			**-2.64**	**[-6.24; 0.96]**	**100.00%**

Heterogeneity: I^2 = 93% [88%; 96%], τ^2 = 15.26

Test for overall effect: p = 0.126

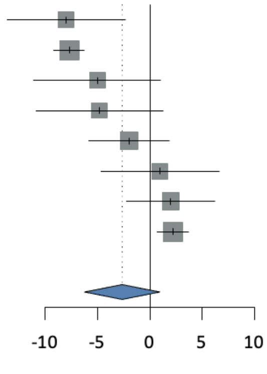

CCP: calcifying chronic pancreatitis, CI: confidence interval, NCCP: non-calcifying chronic pancreatitis, MD: mean difference, SD: standard deviation

Figure S3 Odds of calcification among patients with alcoholic chronic pancreatitis compared to hereditary chronic pancreatitis

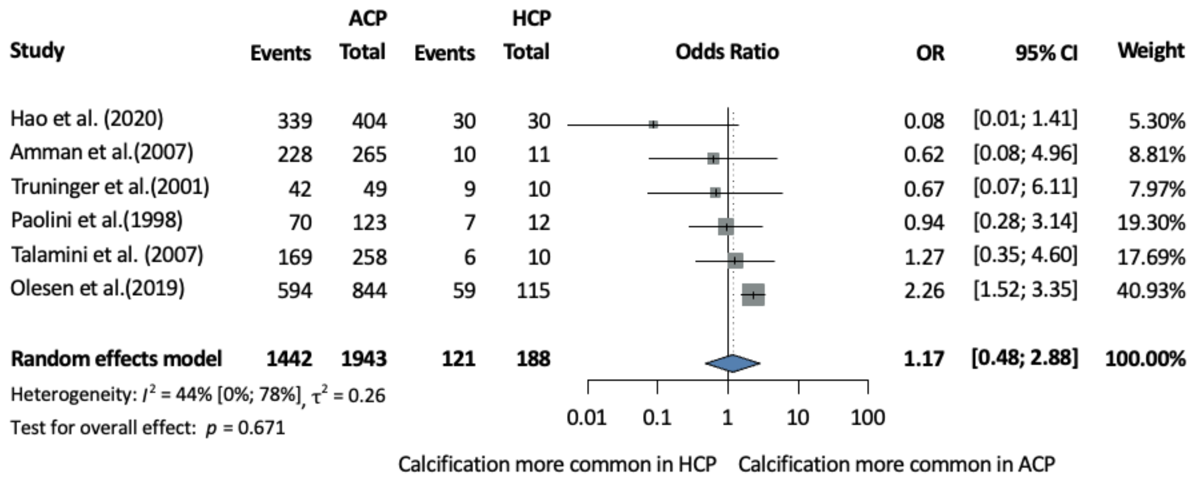

ACP: alcoholic chronic pancreatitis, CI: confidence interval,

HCP: hereditary chronic pancreatitis, OR: odds ratio

Figure S4 Odds of calcification among patients with alcoholic chronic pancreatitis compared to idiopathic chronic pancreatitis

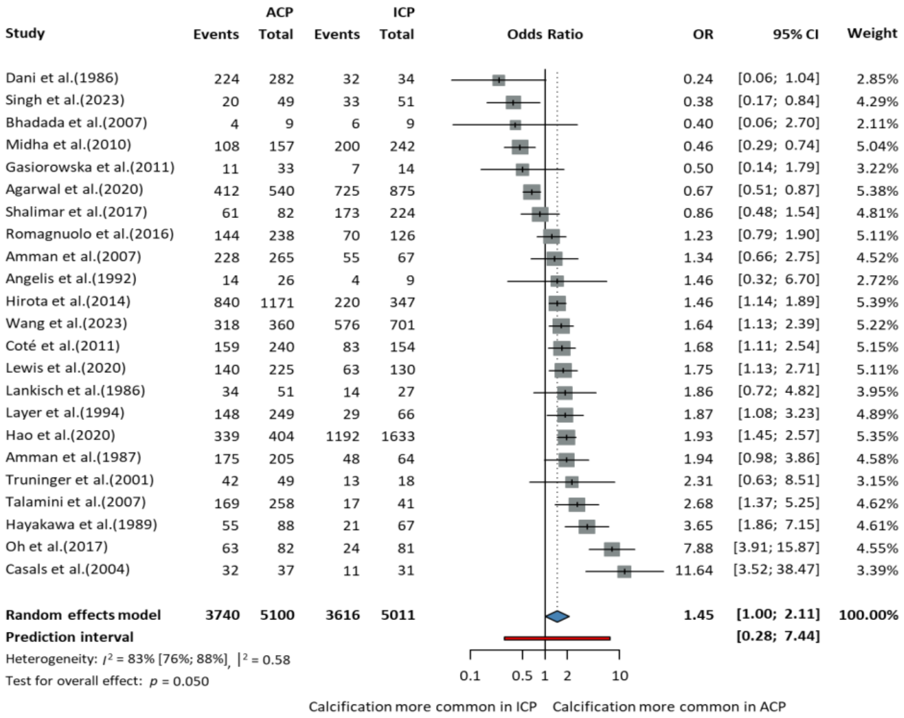

ACP: alcoholic chronic pancreatitis, CI: confidence interval, OR: odds ratio, ICP: idiopathic chronic pancreatitis

Figure S5 Odds of calcification among patients with CFTR mutation compared to patients without CFTR mutation

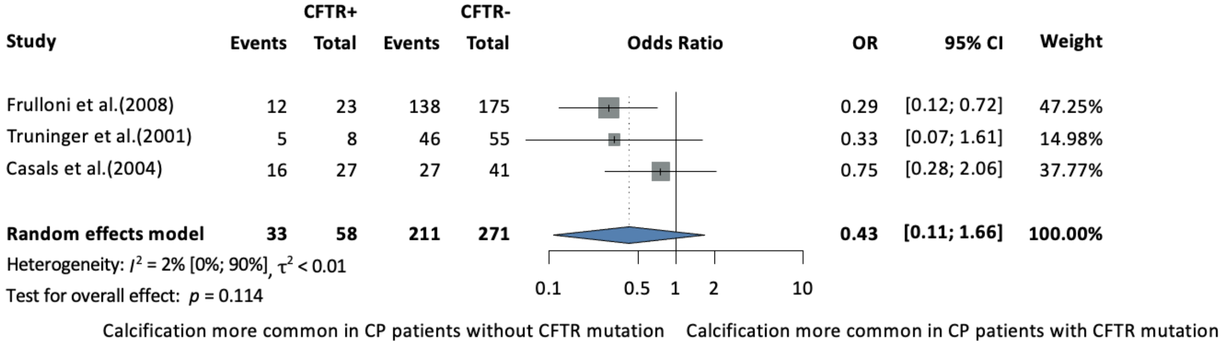

CFTR: Cystic Fibrosis Transmembrane Conductance Regulator, CP: chronic pancreatitis,

CI: confidence interval, OR: odds ratio

Figure S6 Odds of calcification among CP patients with recurrent AP episodes compared to CP patients without recurrent AP episodes

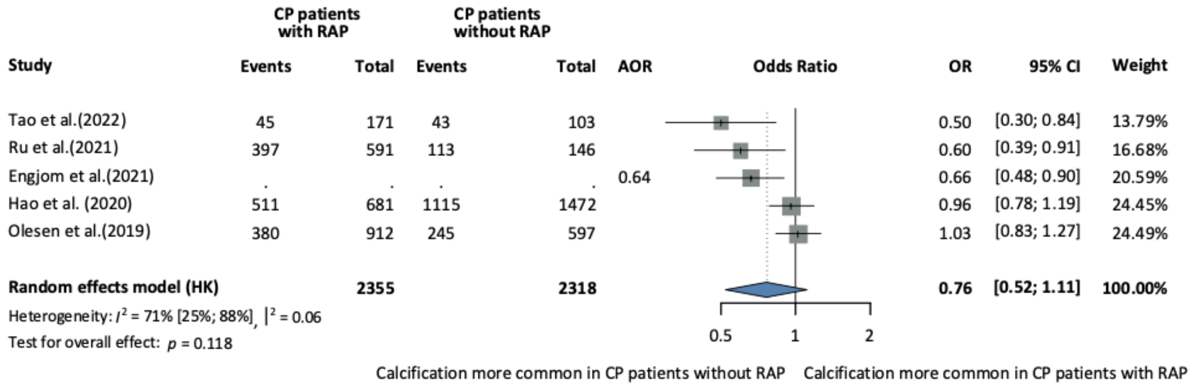

AP: acute pancreatitis, CI: confidence interval, CP: chronic pancreatitis, OR: odds ratio, RAP: recurrent acute pancreatitis

Figure S7 Odds of calcification among AIP patients with recurrent AP episodes compared to AIP patients without recurrent AP episodes

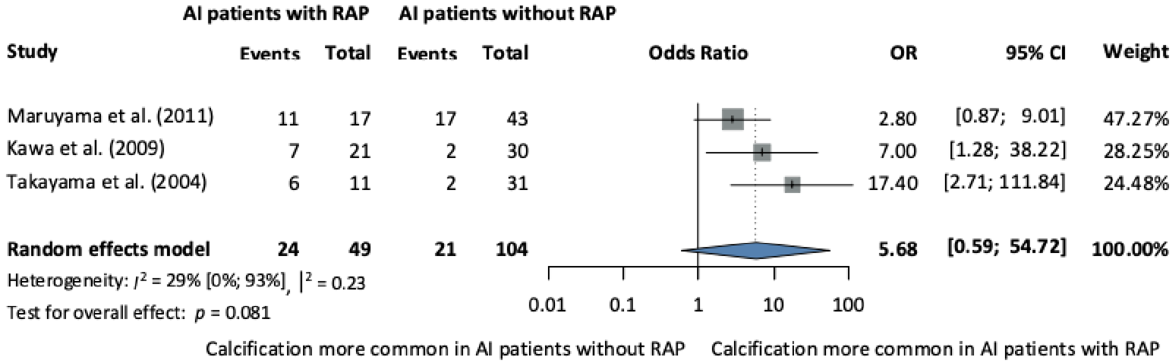

AIP: autoimmune pancreatitis, CI: confidence interval, OR: odds ratio,

RAP: recurrent acute pancreatitis

Figure S8 Odds of calcification among patients with early-onset chronic pancreatitis compared to patients with late-onset chronic pancreatitis

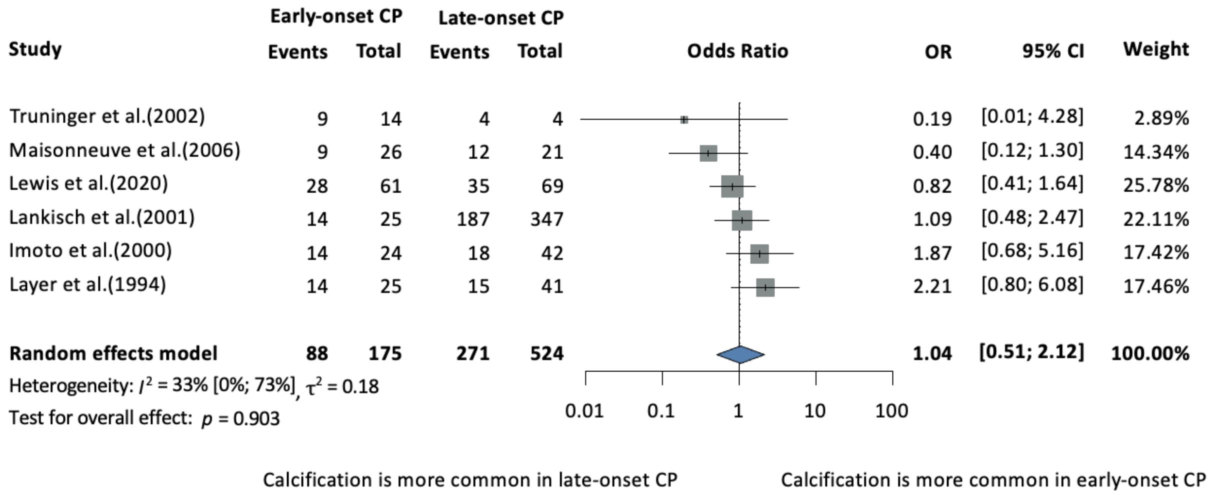

CI: confidence interval, CP: chronic pancreatitis, OR: odds ratio

Figure S9 Mean differences in disease duration between patients with calcifying chronic pancreatitis (CCP) and non-calcifying chronic pancreatitis (NCCP).

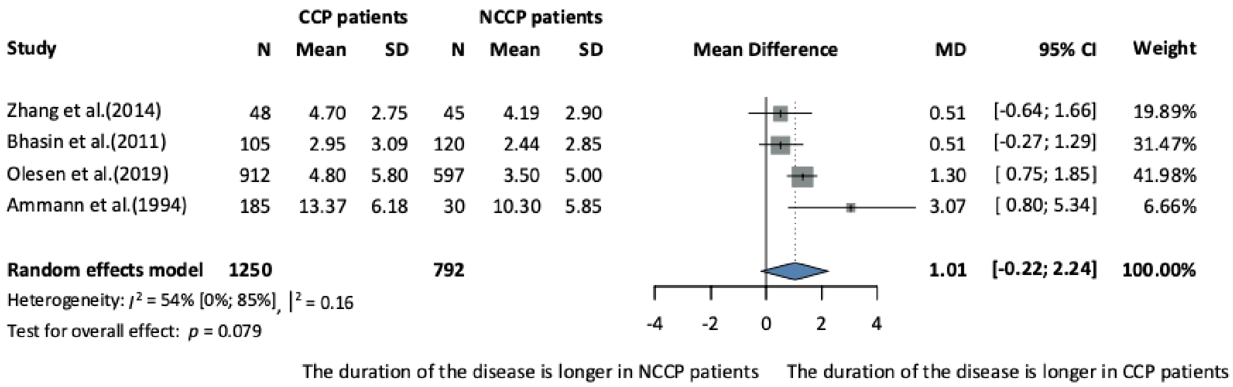

CCP: calcifying chronic pancreatitis, CI: confidence interval, NCCP: non-calcifying chronic pancreatitis, MD: mean differences

Figure S10 Contour-enhanced funnel plot visualizing the odds ratio of calcification in chronic pancreatitis patients who are drinkers compared to non-drinker

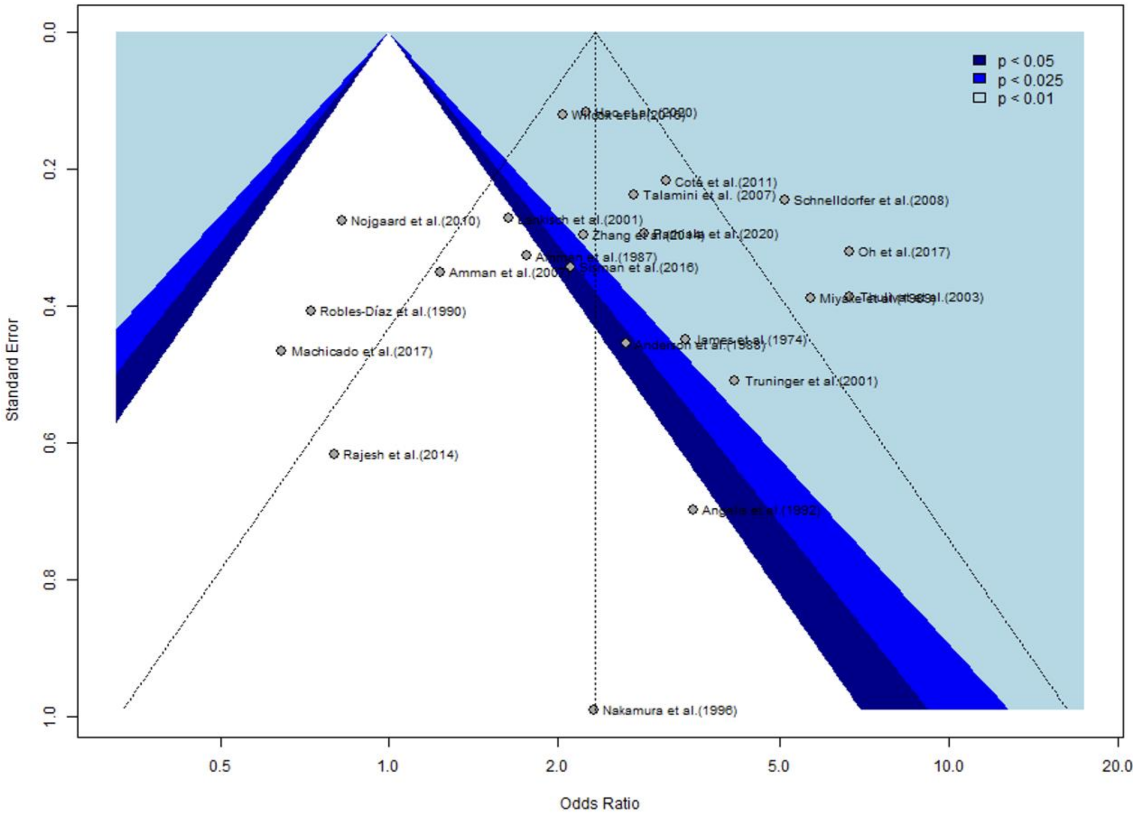

Figure S11 Contour-enhanced funnel plot visualizing the odds ratio of calcification in chronic pancreatitis patients who are smokers compared to non-smokers

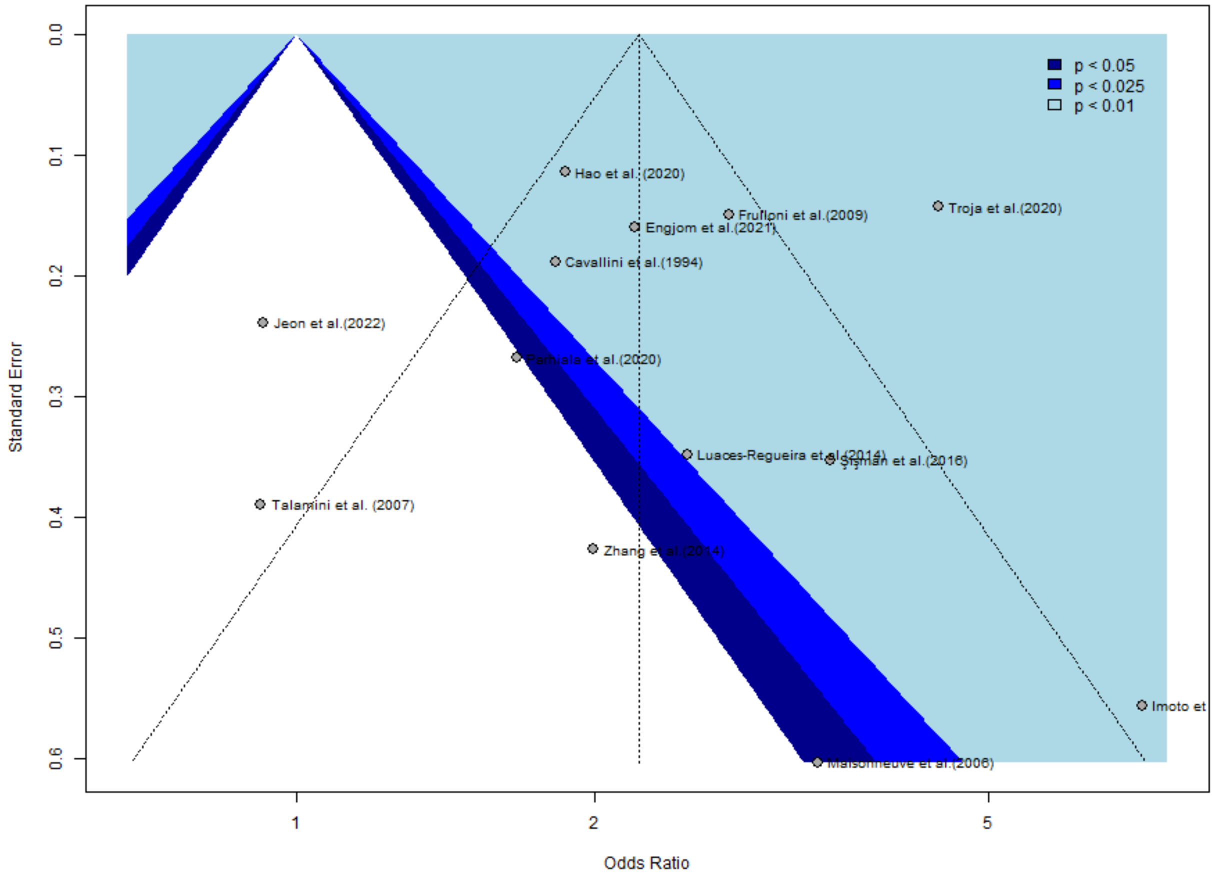

Figure S12 Contour-enhanced funnel plot visualizing the odds ratio of calcification in chronic pancreatitis patients who are women compared to men

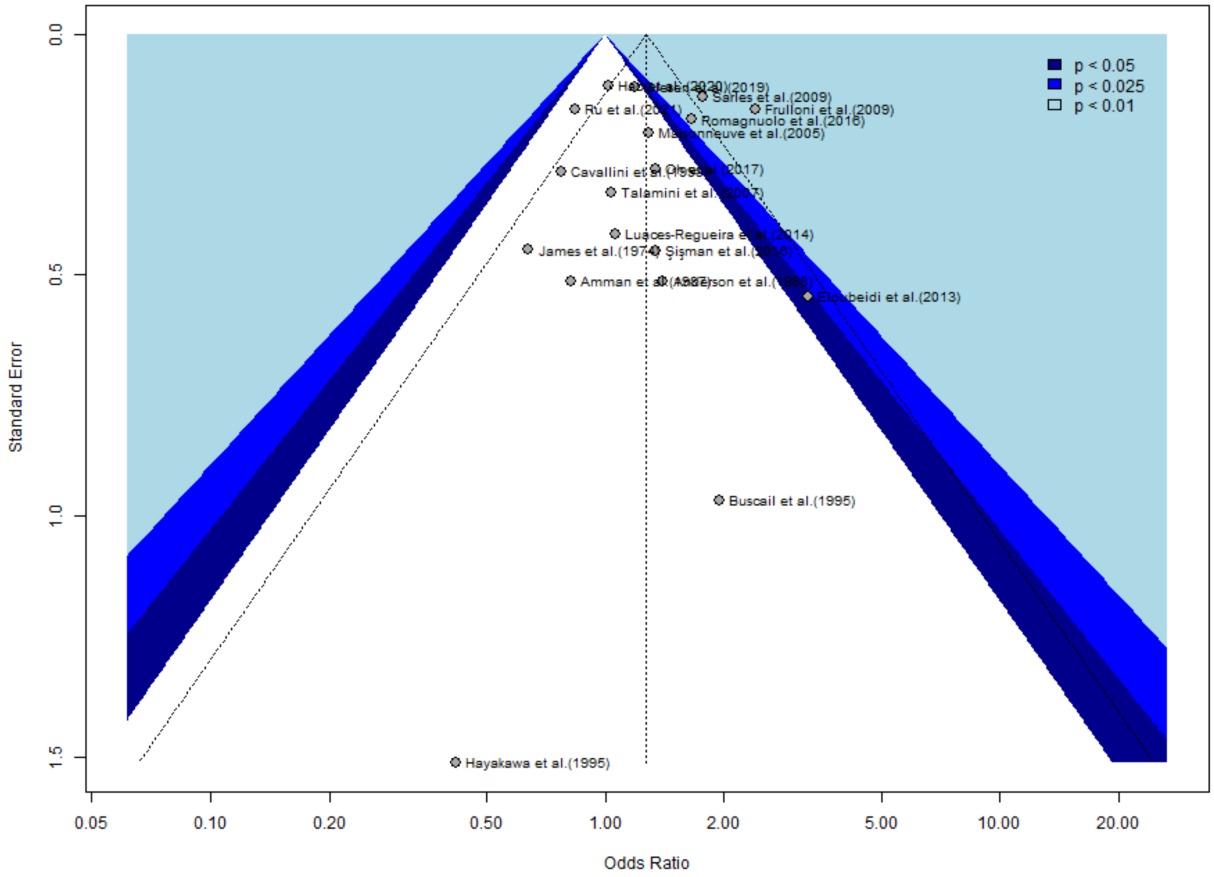

Figure S13 Contour-enhanced funnel plot visualizing the odds ratio of calcification in chronic pancreatitis patients with alcoholic etiology compared to those with idiopathic etiology

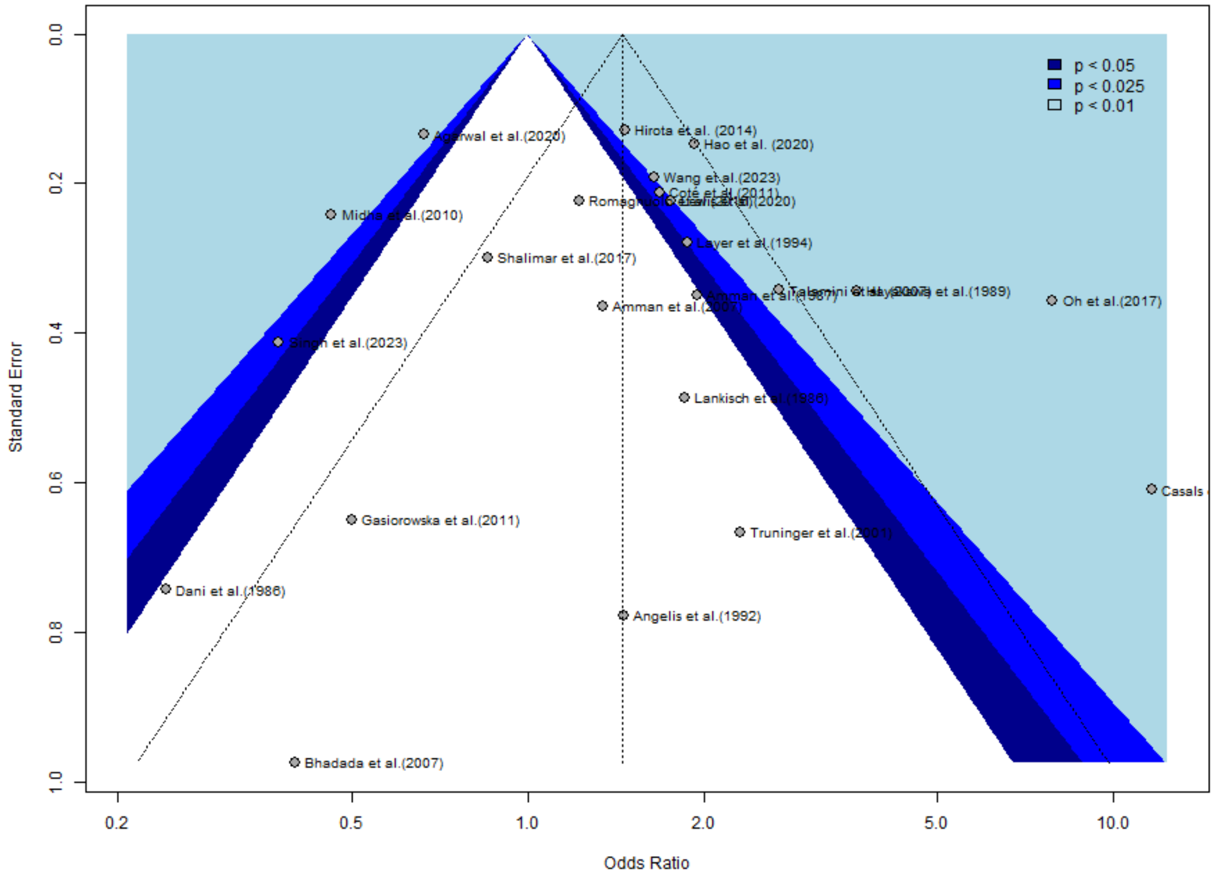

Figure S14A Risk of bias assessment- using the QUIPS tool for chronic pancreatitis etiology as a risk factor for pancreatic calcification

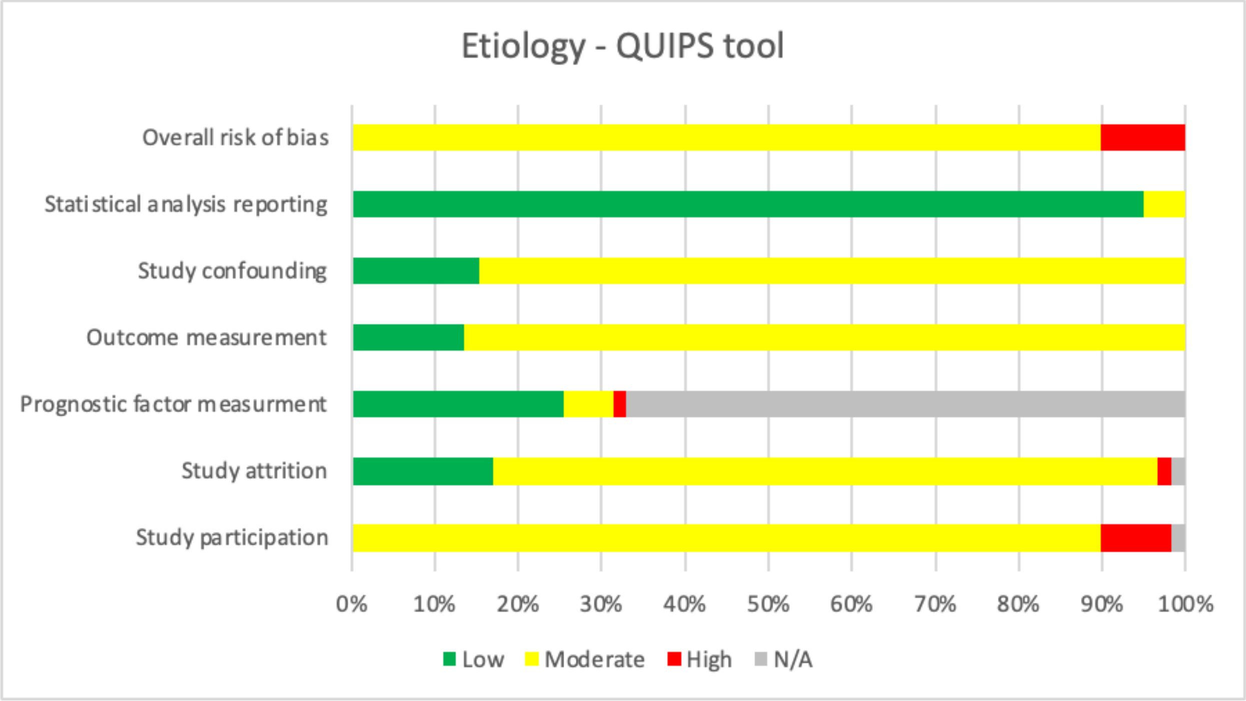

Figure S14B Risk of bias assessment- using the QUIPS tool for age as a risk factor for pancreatic calcification

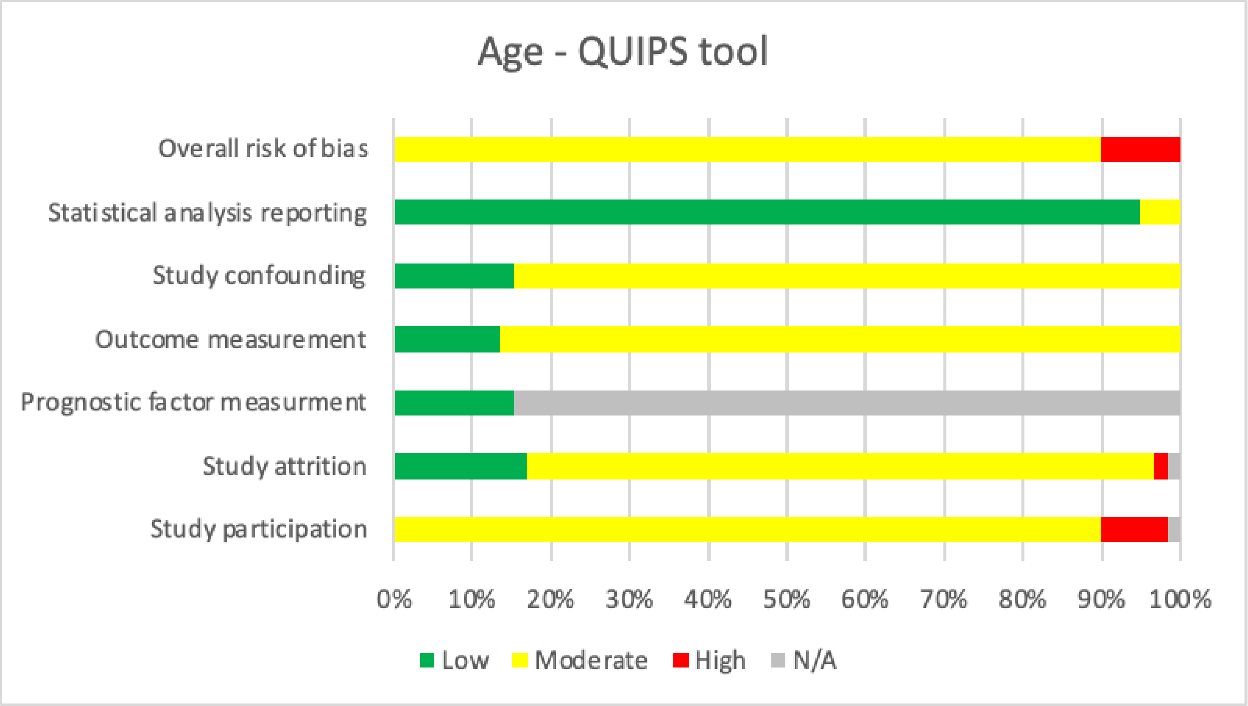

Figure S14C Risk of bias assessment- using the QUIPS tool for drinking habit as a risk factor for pancreatic calcification

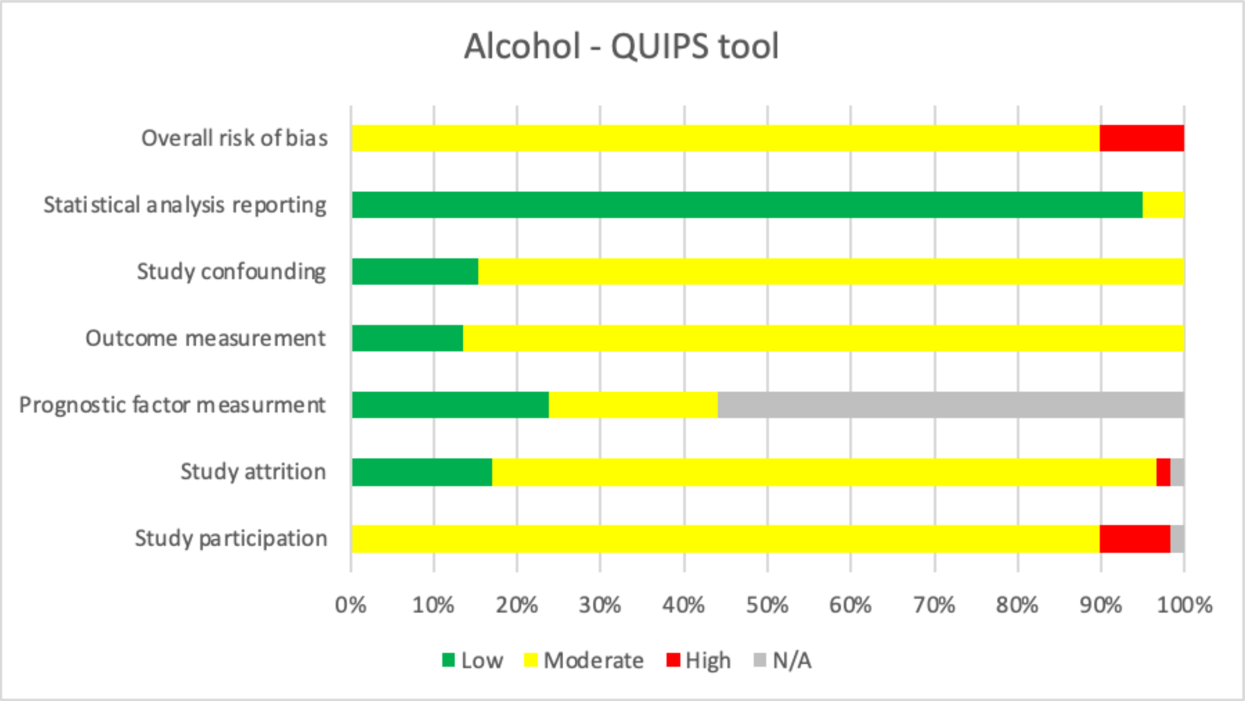

Figure S14D Risk of bias assessment- using the QUIPS tool for smoking habit as a risk factor for pancreatic calcification

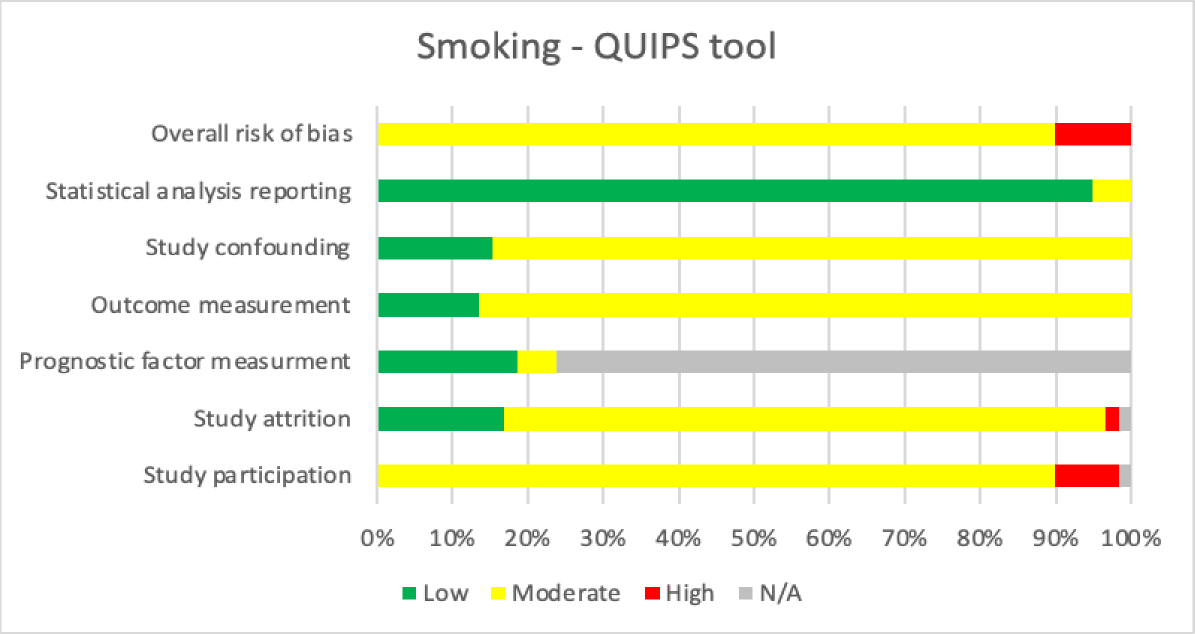

Figure S14E Risk of bias assessment- using the QUIPS tool for sex as a risk factor for pancreatic calcification

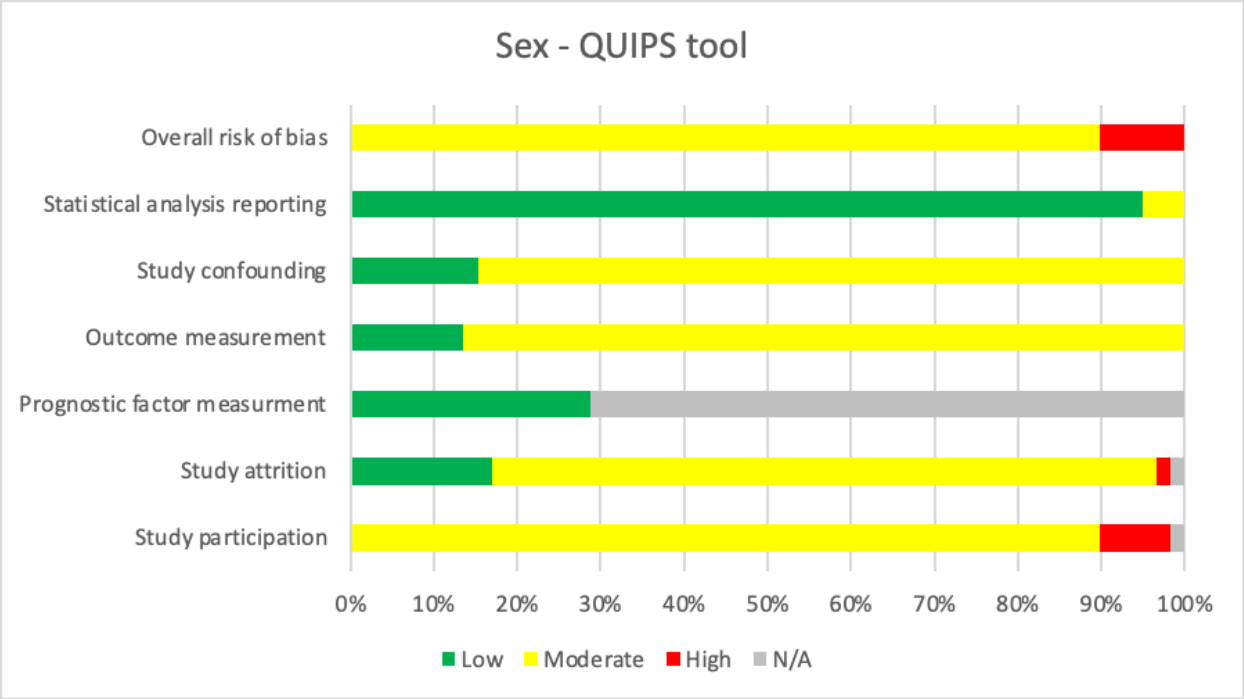

Figure S14F Risk of bias assessment- using the QUIPS tool for CFTR mutation as a risk factor for pancreatic calcification

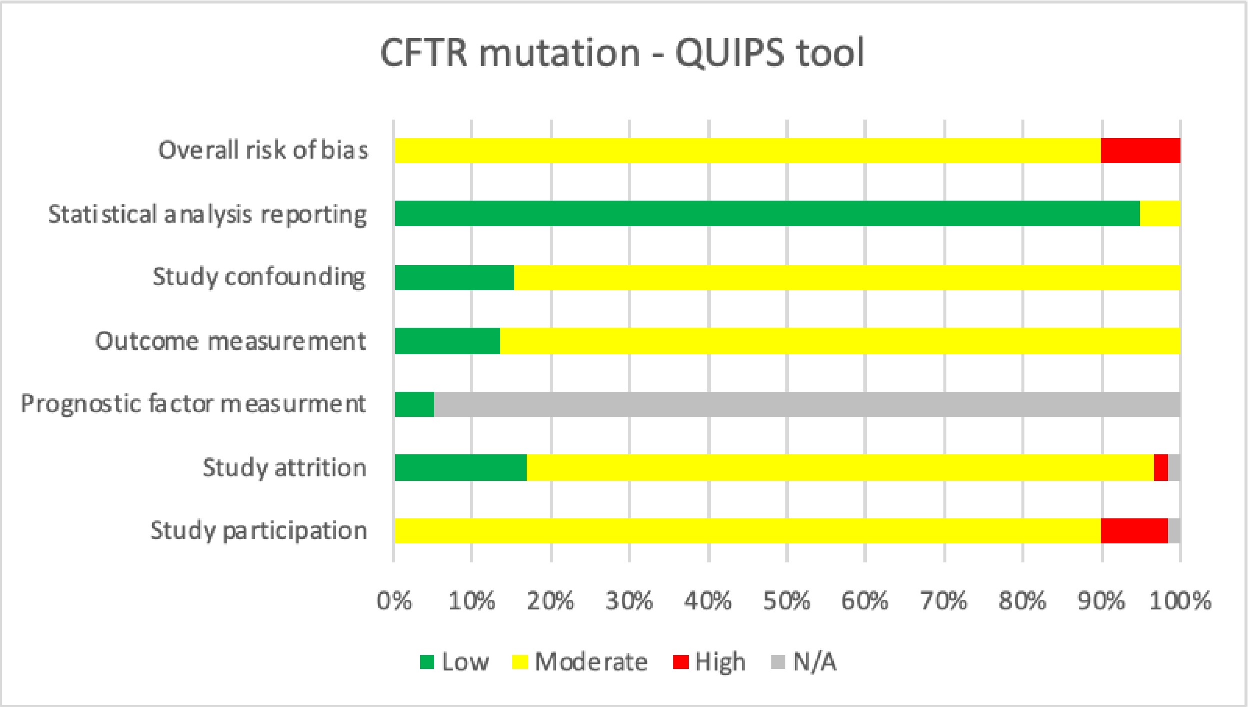

Figure S14G Risk of bias assessment- using the QUIPS tool for RAP as a risk factor for pancreatic calcification

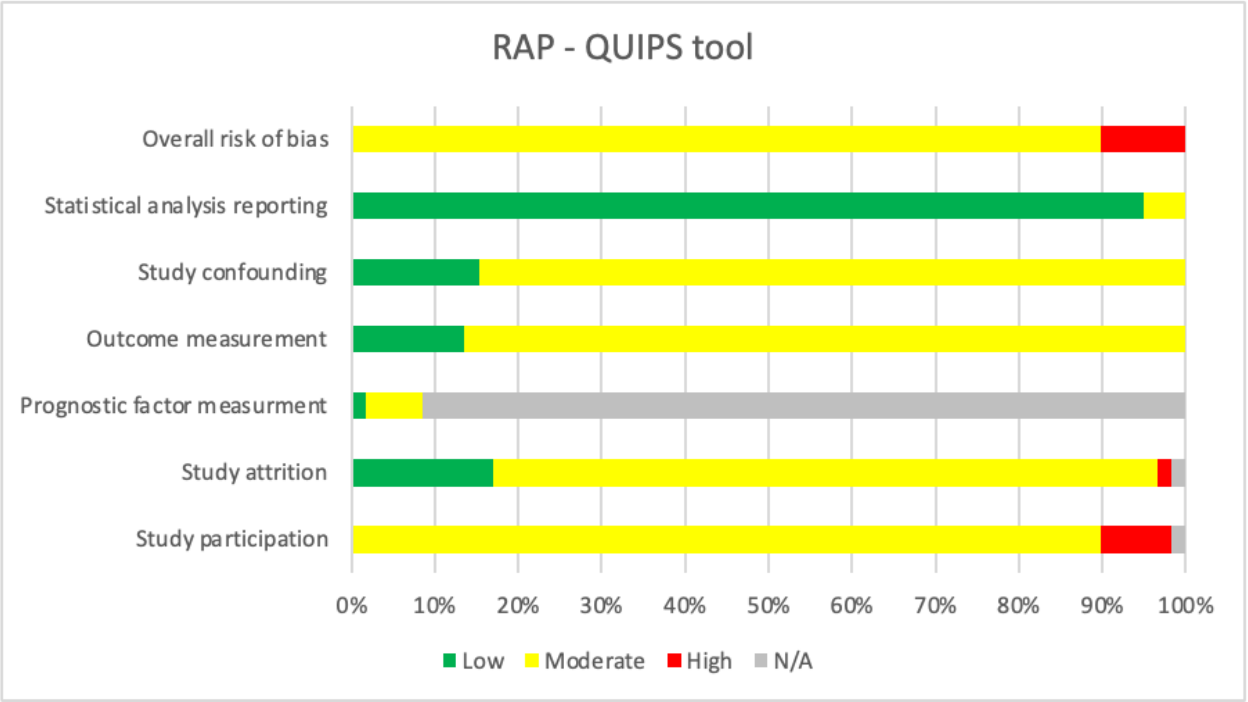

Figure S14H Risk of bias assessment- using the QUIPS tool for duration of CP as a risk factor for pancreatic calcification

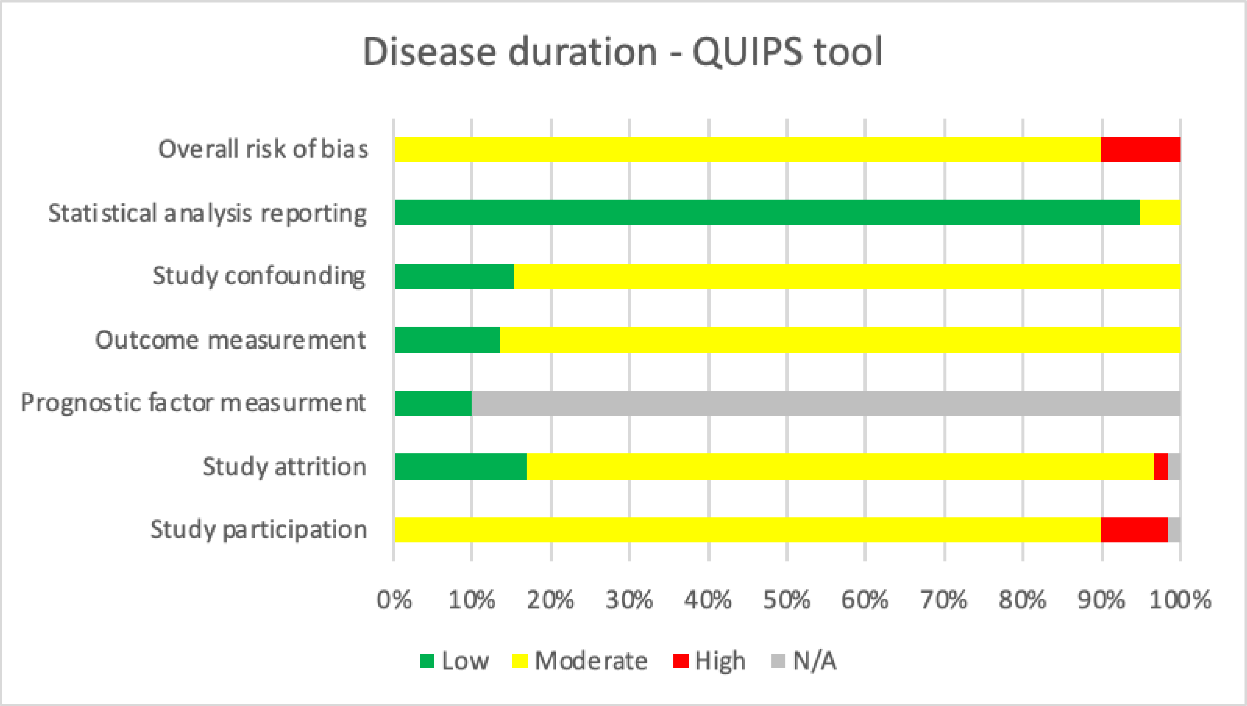

Figure S14I Risk of bias assessment- using the QUIPS tool for onset of chronic pancreatitis as a risk factor for pancreatic calcification

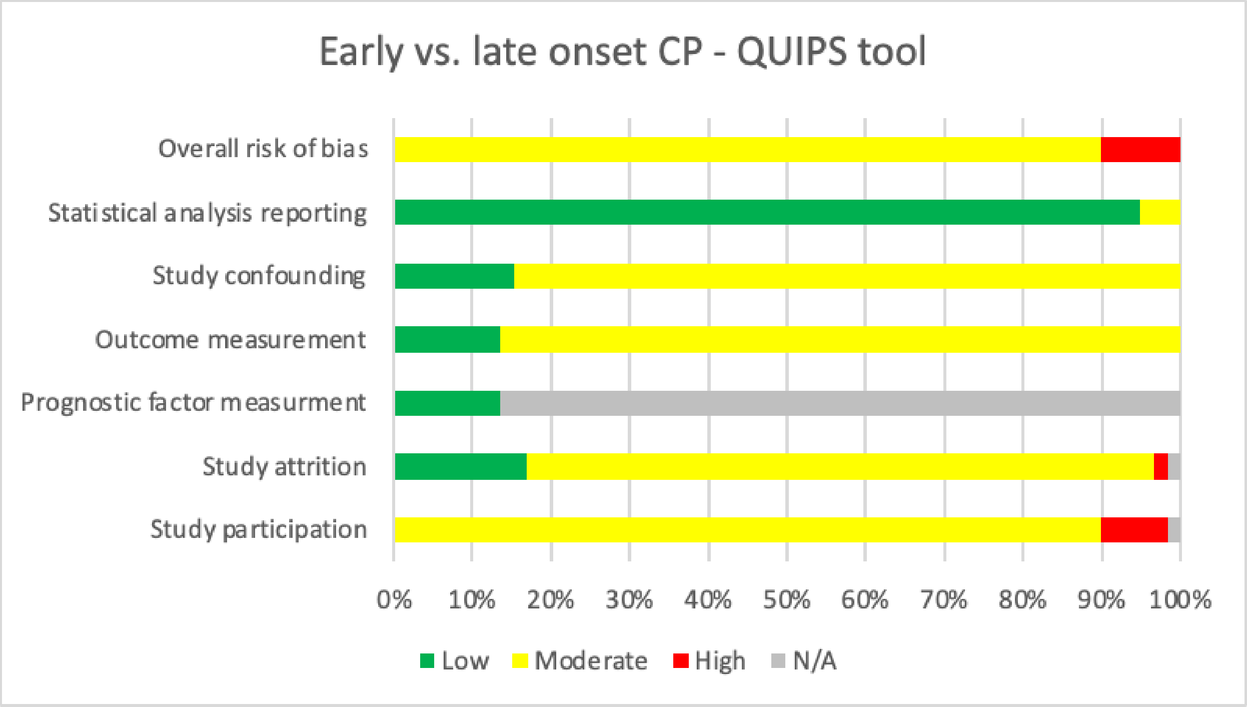

REFERENCES

1. Agarwal, S., et al., *Natural course of chronic pancreatitis and predictors of its progression.* Pancreatology, 2020. **20**(3): p. 347-355.
2. Ammann, R.W., et al., *Differences in the natural history of idiopathic (nonalcoholic) and alcoholic chronic pancreatitis. A comparative long-term study of 287 patients.* Pancreas, 1987. **2**(4): p. 368-77.
3. Ammann, R.W. and B. Mullhaupt, *Do the diagnostic criteria differ between alcoholic and nonalcoholic chronic pancreatitis?* J Gastroenterol, 2007. **42 Suppl 17**: p. 118-26.
4. Ammann, R.W. and B. Muellhaupt, *Progression of alcoholic acute to chronic pancreatitis.* Gut, 1994. **35**(4): p. 552-6.
5. Arvanitakis, M., et al., *Predictive factors for pancreatic cancer in patients with chronic pancreatitis in association with K-ras gene mutation.* Endoscopy, 2004. **36**(6): p. 535-42.
6. Anderson, R.J., et al., *Chronic pancreatitis, HLA and autoimmunity.* Int J Pancreatol, 1988. **3**(1): p. 83-90.
7. De Angelis, C., et al., *Histological study of alcoholic, nonalcoholic, and obstructive chronic pancreatitis.* Pancreas, 1992. **7**(2): p. 193-6.
8. Bhadada, S.K., et al., *Chronic pancreatitis in primary hyperparathyroidism: comparison with alcoholic and idiopathic chronic pancreatitis.* J Gastroenterol Hepatol, 2008. **23**(6): p. 959-64.
9. Bhasin, D.K., et al., *Clinical profile of calcific and noncalcific chronic pancreatitis in north India.* J Clin Gastroenterol, 2011. **45**(6): p. 546-50.
10. Buscail, L., et al., *Endoscopic ultrasonography in chronic pancreatitis: a comparative prospective study with conventional ultrasonography, computed tomography, and ERCP.* Pancreas, 1995. **10**(3): p. 251-7.
11. Casals, T., et al., *Different CFTR mutational spectrum in alcoholic and idiopathic chronic pancreatitis?* Pancreas, 2004. **28**(4): p. 374-9.
12. Cavallini, G., et al., *Effect of alcohol and smoking on pancreatic lithogenesis in the course of chronic pancreatitis.* Pancreas, 1994. **9**(1): p. 42-6.
13. Cavallini, G., et al., *Long-term follow-up of patients with chronic pancreatitis in Italy.* Scand J Gastroenterol, 1998. **33**(8): p. 880-9.

14. Chari, S.T., et al., *Comparative study of the clinical profiles of alcoholic chronic pancreatitis and tropical chronic pancreatitis in Tamil Nadu, south India.* Pancreas, 1992. **7**(1): p. 52-8.

15. Coté, G.A., et al., *Alcohol and smoking as risk factors in an epidemiology study of patients with chronic pancreatitis.* Clin Gastroenterol Hepatol, 2011. **9**(3): p. 266-73; quiz e27.

16. Dani, R., F.J. Penna, and C.E. Nogueira, *Etiology of chronic calcifying pancreatitis in Brazil: a report of 329 consecutive cases.* Int J Pancreatol, 1986. **1**(5-6): p. 399-406.

17. Dítě, P., et al., *The role of chronic inflammation: chronic pancreatitis as a risk factor of pancreatic cancer.* Dig Dis, 2012. **30**(3): p. 277-83.

18. Ectors, N., et al., *Non-alcoholic duct destructive chronic pancreatitis.* Gut, 1997. **41**(2): p. 263-8.

19. Eloubeidi, M.A., et al., *Ratio of pancreatic duct caliber to width of pancreatic gland by endosonography is predictive of pancreatic cancer.* Pancreas, 2013. **42**(4): p. 670-9.

20. Engjom, T., et al., *Aetiological risk factors are associated with distinct imaging findings in patients with chronic pancreatitis: A study of 959 cases from the Scandinavian Baltic Pancreatic Club (SBPC) imaging database.* Pancreatology, 2021. **21**(4): p. 688-697.

21. Frulloni, L., et al., *Clinical and radiological outcome of patients suffering from chronic pancreatitis associated with gene mutations.* Pancreas, 2008. **37**(4): p. 371-6.

22. Frulloni, L., et al., *Chronic pancreatitis: report from a multicenter Italian survey (PanCroInfAISP) on 893 patients.* Dig Liver Dis, 2009. **41**(4): p. 311-7.

23. Gasiorowska, A., et al., *The prevalence of cationic trypsinogen (PRSS1) and serine protease inhibitor, Kazal type 1 (SPINK1) gene mutations in Polish patients with alcoholic and idiopathic chronic pancreatitis.* Dig Dis Sci, 2011. **56**(3): p. 894-901.

24. Hao, L., et al., *Risk Factors and Nomogram for Pancreatic Stone Formation in Chronic Pancreatitis over a Long-Term Course: A Cohort of 2,153 Patients.* Digestion, 2020. **101**(4): p. 473-483.

25. Hayakawa, T., et al., *Pancreatic stone protein and lactoferrin in human pancreatic juice in chronic pancreatitis.* Pancreas, 1995. **10**(2): p. 137-42.

26. Hayakawa, T., et al., *Chronic alcoholism and evolution of pain and prognosis in chronic pancreatitis.* Dig Dis Sci, 1989. **34**(1): p. 33-8.

27. Hirota, M., et al., *The seventh nationwide epidemiological survey for chronic pancreatitis in Japan: clinical significance of smoking habit in Japanese patients.* Pancreatology, 2014. **14**(6): p. 490-6.

28. Hirth, M., et al., *Clinical Course of Chronic Pancreatitis in Elderly Patients.* Digestion, 2019. **100**(3): p. 152-159.

29. Imoto, M. and E.P. DiMagno, *Cigarette smoking increases the risk of pancreatic calcification in late-onset but not early-onset idiopathic chronic pancreatitis.* Pancreas, 2000. **21**(2): p. 115-9.

30. James, O., J.E. Agnew, and I.A. Bouchier, *Chronic pancreatitis in England: a changing picture?* Br Med J, 1974. **2**(5909): p. 34-8.

31. Jeon, C.Y., et al., *Lifetime smoking history and cohort-based smoking prevalence in chronic pancreatitis.* Pancreatology, 2021.

32. Kamisawa, T., et al., *Chronic pancreatitis in the elderly in Japan.* Pancreatology, 2004. **4**(3-4): p. 223-7; discussion 227-8.

33. Kanai, K., et al., *Autoimmune Pancreatitis Can Transform Into Chronic Features Similar to Advanced Chronic Pancreatitis With Functional Insufficiency Following Severe Calcification.* Pancreas, 2016. **45**(8): p. 1189-95.

34. Kawa, S., et al., *HLA DRB10405-DQB10401 haplotype is associated with autoimmune pancreatitis in the Japanese population.* Gastroenterology, 2002. **122**(5): p. 1264-9.

35. Kawa, S., et al., *Long-term follow-up of autoimmune pancreatitis: characteristics of chronic disease and recurrence.* Clin Gastroenterol Hepatol, 2009. **7**(11 Suppl): p. S18-22.

36. Keim, V., et al., *The course of genetically determined chronic pancreatitis.* Jop, 2003. **4**(4): p. 146-54.

37. Kuraishi, Y., et al., *Corticosteroids prevent the progression of autoimmune pancreatitis to chronic pancreatitis.* Pancreatology, 2020. **20**(6): p. 1062-1068.

38. Lankisch, P.G., et al., *Pancreatic calcifications: no indicator of severe exocrine pancreatic insufficiency.* Gastroenterology, 1986. **90**(3): p. 617-21.

39. Lankisch, M.R., et al., *The effect of small amounts of alcohol on the clinical course of chronic pancreatitis.* Mayo Clin Proc, 2001. **76**(3): p. 242-51.

40. Layer, P., et al., *The different courses of early- and late-onset idiopathic and alcoholic chronic pancreatitis.* Gastroenterology, 1994. **107**(5): p. 1481-7.

41. Law, R., et al., *Cigarette smoking is independently associated with chronic pancreatitis.* Pancreatology, 2010. **10**(1): p. 54-9.

42. Lee, J.W., et al., *Association between Smoking and the Progression of Computed Tomography Findings in Chronic Pancreatitis.* Gut Liver, 2016. **10**(3): p. 464-9.

43. Lewis, M.D., et al., *Differences in Age at Onset of Symptoms, and Effects of Genetic Variants, in Patients With Early vs Late-Onset Idiopathic Chronic Pancreatitis in a North American Cohort.* Clin Gastroenterol Hepatol, 2021. **19**(2): p. 349-357.

44. Luaces-Regueira, M., et al., *Smoking as a risk factor for complications in chronic pancreatitis.* Pancreas, 2014. **43**(2): p. 275-80.

45. Machicado, J.D., et al., *A population-based evaluation of the natural history of chronic pancreatitis.* Pancreatology, 2018. **18**(1): p. 39-45.

46. Maisonneuve, P., et al., *Cigarette smoking accelerates progression of alcoholic chronic pancreatitis.* Gut, 2005. **54**(4): p. 510-4.

47. Maisonneuve, P., et al., *Impact of smoking on patients with idiopathic chronic pancreatitis.* Pancreas, 2006. **33**(2): p. 163-8.

48. Maruyama, M., et al., *Risk factors for pancreatic stone formation in autoimmune pancreatitis over a long-term course.* J Gastroenterol, 2012. **47**(5): p. 553-60.

49. Midha, S., et al., *Idiopathic chronic pancreatitis in India: phenotypic characterisation and strong genetic susceptibility due to SPINK1 and CFTR gene mutations.* Gut, 2010. **59**(6): p. 800-7.

50. Miyake, H., et al., *Prognosis and prognostic factors in chronic pancreatitis.* Dig Dis Sci, 1989. **34**(3): p. 449-55.

51. Müllhaupt, B., K. Truninger, and R. Ammann, *Impact of etiology on the painful early stage of chronic pancreatitis: a long-term prospective study.* Z Gastroenterol, 2005. **43**(12): p. 1293-301.

52. Nakamura, T., et al., *Correlation between pancreatic endocrine and exocrine function and characteristics of pancreatic endocrine function in patients with diabetes mellitus owing to chronic pancreatitis.* Int J Pancreatol, 1996. **20**(3): p. 169-75.

53. Nøjgaard, C., et al., *Danish patients with chronic pancreatitis have a four-fold higher mortality rate than the Danish population.* Clin Gastroenterol Hepatol, 2010. **8**(4): p. 384-90.

54. Oh, H.C., et al., *Low Serum Pancreatic Amylase and Lipase Values Are Simple and Useful Predictors to Diagnose Chronic Pancreatitis.* Gut Liver, 2017. **11**(6): p. 878-883.

55. Olesen, S.S., et al., *Pancreatic calcifications associate with diverse aetiological risk factors in patients with chronic pancreatitis: A multicentre study of 1500 cases.* Pancreatology, 2019. **19**(7): p. 922-928.

56. Paolini, O., et al., *The natural history of hereditary chronic pancreatitis: a study of 12 cases compared to chronic alcoholic pancreatitis.* Pancreas, 1998. **17**(3): p. 266-71.

57. Parhiala, M., J. Sand, and J. Laukkarinen, *A population-based study of chronic pancreatitis in Finland: Effects on quality of life.* Pancreatology, 2020. **20**(3): p. 338-346.

58. Rajesh, G., et al., *Clinical profile of early-onset and late-onset idiopathic chronic pancreatitis in South India.* Indian J Gastroenterol, 2014. **33**(3): p. 231-6.

59. Rebours, V., et al., *Smoking and the course of recurrent acute and chronic alcoholic pancreatitis: a dose-dependent relationship.* Pancreas, 2012. **41**(8): p. 1219-24.

60. Regunath, H., et al., *Anthropometric measurements of nutritional status in chronic pancreatitis in India: comparison of tropical and alcoholic pancreatitis.* Indian J Gastroenterol, 2011. **30**(2): p. 78-83.

61. Robles-Díaz, G., et al., *Chronic pancreatitis in Mexico City.* Pancreas, 1990. **5**(4): p. 479-83.

62. Romagnuolo, J., et al., *Clinical Profile, Etiology, and Treatment of Chronic Pancreatitis in North American Women: Analysis of a Large Multicenter Cohort.* Pancreas, 2016. **45**(7): p. 934-40.

63. Ru, N., et al., *Factors associated with prior acute pancreatitis episodes among patients with chronic pancreatitis.* Dig Liver Dis, 2021. **53**(9): p. 1148-1153.

64. Sarles, H., R.C. Cros, and J.M. Bidart, *A multicenter inquiry into the etiology of pancreatic diseases.* Digestion, 1979. **19**(2): p. 110-25.

65. Schnelldorfer, T. and D.B. Adams, *Surgical treatment of alcohol-associated chronic pancreatitis: the challenges and pitfalls.* Am Surg, 2008. **74**(6): p. 503-7; discussion 508-9.

66. Shalimar, et al., *Long-term pain relief with optimized medical treatment including antioxidants and step-up interventional therapy in patients with chronic pancreatitis.* J Gastroenterol Hepatol, 2017. **32**(1): p. 270-277.

67. Sinha, A., et al., *Predictors of Post-Operative Pain Relief in Patients with Chronic Pancreatitis Undergoing the Frey or Whipple Procedure.* J Gastrointest Surg, 2016. **20**(4): p. 734-40.

68. Singh, N., et al., *Genetic polymorphisms in phase II metabolizing enzymes in alcoholic and idiopathic chronic pancreatitis: Indian scenario.* Indian J Gastroenterol, 2023. **42**(2): p. 199-208.

69. Şişman, G., et al., *Demographic characteristics of chronic pancreatitis patients in the era of endosonography: Experience of a single tertiary referral center in Turkey.* Turk J Gastroenterol, 2016. **27**(3): p. 284-9.

70. Takayama, M., et al., *Recurrent attacks of autoimmune pancreatitis result in pancreatic stone formation.* Am J Gastroenterol, 2004. **99**(5): p. 932-7.

71. Talamini, G., et al., *Smoking cessation at the clinical onset of chronic pancreatitis and risk of pancreatic calcifications.* Pancreas, 2007. **35**(4): p. 320-6.

72. Tao, H., et al., *Clinical Characteristics of Patients With Chronic Pancreatitis With or Without Prior Acute Pancreatitis Are Different.* Pancreas, 2022. **51**(8): p. 950-956.

73. Thuluvath, P.J., et al., *Chronic pancreatitis. Long-term pain relief with or without surgery, cancer risk, and mortality.* J Clin Gastroenterol, 2003. **36**(2): p. 159-65.

74. Tjora, E., et al., *Patient reported exposure to smoking and alcohol abuse are associated with pain and other complications in patients with chronic pancreatitis.* Pancreatology, 2020. **20**(5): p. 844-851.

75. Truninger, K., et al., *Mutations of the cystic fibrosis gene in patients with chronic pancreatitis.* Am J Gastroenterol, 2001. **96**(9): p. 2657-61.

76. Wang, Q., et al., *Eosinophilia associated with chronic pancreatitis.* Pancreas, 2009. **38**(2): p. 149-53.

77. Wang, Y.C., et al., *High Clinical and Genetic Similarity Between Chronic Pancreatitis Associated With Light-to-Moderate Alcohol Consumption and Classical Alcoholic Chronic Pancreatitis.* Gastro Hep Adv, 2023. **2**(2): p. 186-195.

78. Wilcox, C.M., et al., *Racial Differences in the Clinical Profile, Causes, and Outcome of Chronic Pancreatitis.* Am J Gastroenterol, 2016. **111**(10): p. 1488-1496.

79. Zhang, G.W., et al., *Analysis of risk factors for pancreatic duct stones formation in patients with alcoholic chronic pancreatitis.* Pancreatology, 2014. **14**(2): p. 109-13.

80. Zou, W.B., et al., *SPINK1, PRSS1, CTRC, and CFTR Genotypes Influence Disease Onset and Clinical Outcomes in Chronic Pancreatitis.* Clin Transl Gastroenterol, 2018. **9**(11): p. 204.
